# Population differences in host plant preference and the importance of yeast and plant substrate to volatile composition

**DOI:** 10.1002/ece3.2993

**Published:** 2017-04-18

**Authors:** Priya Date, Amber Crowley‐Gall, Aaron F. Diefendorf, Stephanie M. Rollmann

**Affiliations:** ^1^ Department of Biological SciencesUniversity of CincinnatiCincinnatiOHUSA; ^2^ Department of GeologyUniversity of CincinnatiCincinnatiOHUSA; ^3^ Present address: Department of PediatricsYale University School of MedicineNew HavenCT06520USA

**Keywords:** behavior, *Drosophila*, evolution, olfaction, performance, volatile

## Abstract

Divergent selection between environments can result in changes to the behavior of an organism. In many insects, volatile compounds are a primary means by which host plants are recognized and shifts in plant availability can result in changes to host preference. Both the plant substrate and microorganisms can influence this behavior, and host plant choice can have an impact on the performance of the organism. In *Drosophila mojavensis,* four geographically isolated populations each use different cacti as feeding and oviposition substrates and identify those cacti by the composition of the volatile odorants emitted. Behavioral tests revealed *D. mojavensis* populations vary in their degree of preference for their natural host plant. Females from the Mojave population show a marked preference for their host plant, barrel cactus, relative to other cactus choices. When flies were given a choice between cacti that were not their host plant, the preference for barrel and organ pipe cactus relative to agria and prickly pear cactus was overall lower for all populations. Volatile headspace composition is influenced by the cactus substrate, microbial community, and substrate‐by‐microorganism interactions. Differences in viability, developmental time, thorax length, and dry body weight exist among populations and depend on cactus substrate and population‐by‐cactus interactions. However, no clear association between behavioral preference and performance was observed. This study highlights a complex interplay between the insect, host plant, and microbial community and the factors mediating insect host plant preference behavior.

## 1 Introduction

Divergent selection between different ecological environments is often mirrored by adaptive changes in the morphology (Hoekstra, Drumm, & Nachman, 2004; Sandoval & Crespi, 2008) and/or behavior of organisms (Etges, 1998; Funk & Bernays, 2001; Schmidt, Matzkin, Ippolito, & Eanes, 2007). Environmentally mediated changes to behavior are frequently associated with changes in the sensory systems that help mediate the behavior (Fischer, Soares, Archer, Ghalambor, & Hoke, 2013; Linz et al., 2013; Miyagi et al., 2012). In many insects, for example, host preference behavior relies on olfaction for host plant recognition. Blends of specific volatiles as well as single compounds can relay information about oviposition and feeding resources, thereby influencing host preference (Dweck et al., 2013; Linn et al., 2003; Riffell, Lei, & Hildebrand, 2009). Moreover, shifts in neurophysiological sensitivity to such volatiles have been shown to influence behavior both within and between species (Date et al., 2013; Dekker, Ibba, Siju, Stensmyr, & Hansson, 2006; Olsson, Linn, & Roelofs, 2006).

*Drosophila* is well suited for examining the determinants of host preference behavior. This behavior involves a complex interplay between the insect, host plant, and microbial community. More specifically, microorganisms, such as yeast, are a nutritional source for the organism, and also detoxify harmful plant compounds, resulting in a suitable environment for larval development (Starmer & Aberdeen, 1990; Starmer & Fogleman, 1986). In addition, volatiles emitted during this fermentation process are used by the insects for appropriate host plant identification and the insects themselves can act as vectors impacting microbial distributions (Gilbert, 1980; Starmer, 1982; Fogleman & Foster, 1989). Therefore, the relationship between plant substrate, microorganism, and insect can have direct consequences on the fitness of the organism, on host preference behavior, and ultimately divergence among populations (Biere & Tack, 2013; Fogleman & Danielson, 2001; Janson, Stireman, Singer, & Patrick, 2008; Starmer & Fogleman, 1986; Sugio, Dubreuil, Giron, & Simon, 2015).

The cactophilic fly, *Drosophila mojavensis,* is a model for understanding determinants of host preference and is an excellent example of insect–microorganism–plant relationships (Downing, 1985; Fogleman & Danielson, 2001). *Drosophila mojavensis* is composed of four geographically isolated populations in the southwestern United States and Mexico. The ancestral Baja and mainland Sonoran populations are hypothesized to have diverged 230,000–270,000 years ago, with the subsequent divergence of the Sonoran Desert and Mojave populations 117,000–135,000 years ago (Smith, Lohse, Etges, & Ritchie, 2012). Populations in Baja, the Sonoran Desert, Mojave Desert, and Santa Catalina Island each feed, mate, and oviposit on different cacti: agria (*Stenocereus gummosus*), organ pipe (*Stenocereus thurberi*), barrel (*Ferocactus cylindraceus*), or prickly pear (*Opuntia sp*.), respectively (Heed, 1982). These host cacti vary in their volatile compositions, as a result of the by‐products produced during plant–microbe interaction during the fermentation process, with the volatiles being a primary cue for host recognition (Date et al., 2013; Downing, 1985; Fogleman & Abril, 1990; Newby & Etges, 1998; Wright & Setzer, 2014). Cactus chemical composition and the yeast species found on these necrotic cacti have been fairly well studied, particularly in the Baja and Sonoran Desert regions (Fogleman, Heed, & Kircher, 1982; Foster & Fogleman, 1994; Kircher, 1982). Moreover, Newby & Etges, 1998 examined behavioral responses to synthetic volatile mixtures for a subset of the populations and cacti. This study showed that these populations varied in their attraction to host‐specific synthetic mixtures of agria and organ pipe cactus but, overall, preferred agria mixtures. However, Date et al., 2013 recently showed that the Mojave population has pronounced alterations in electrophysiological responses of the olfactory sensory organs that support a shift of their olfactory system toward recognition of their host plant, barrel cactus. Additionally, a synthetic mixture of barrel volatiles was shown to preferentially attract flies from the Mojave population (Date et al., 2013). Population differences in olfactory sensory neuron number, sensitivity, and specificity have also been observed in this system (Crowley‐Gall et al., 2016).

A thorough understanding of the determinants of host preference, however, requires a systematic examination of the patterns of olfactory preference and performance in all four *D. mojavensis* populations for all four cacti. Previous studies were limited in their scope, and among other things, the relative importance of substrate and microorganism to host plant volatile composition merits more complete investigation. Here, we measure population differences in olfactory preferences, assess the importance of microorganisms to the volatile composition of plant substrates, and evaluate the effects of host plant substrate on fly performance with the long‐term goal of unraveling key factors underlying host preference in this system.

## 2 Materials and methods

### 2.1 *Drosophila* stocks

Flies were obtained from the *Drosophila* Species Stock Center or kindly provided by Dr. Bill Etges and are as follows: Baja California population [San Quintin (SQ59a)]; the mainland Sonoran population (Organ Pipe National Monument, Arizona [OPNM9]); the Mojave population (Providence Mountain, CA [A997b]); and Santa Catalina Island population [stock number 15081‐1352.22]. All flies were reared on cactus–banana–agar medium and were maintained at 25°C, under a 12‐hr L/D cycle.

### 2.2 Cactus fermentation

For host preference tests, 70 g of tissue from each of the four cacti was heat‐sterilized (e.g., Etges & de Oliveira, 2014; Etges & Heed, 1987) and placed in a sterile glass jar. At room temperature, each tissue sample was inoculated with a 1.0‐ml mixture of seven yeast species (*Pichia cactophila, Pichia mexicana, Starmera amethionina, Candida valida, Candida sonorensis, Dipodascus starmeri, and Sporopachydermia cereana*) and 0.5 ml of one pectolytic bacterium *Erwinia cacticida*. These species have been documented to be present on necrotic cacti and used previously in *D. mojavensis* rearing experiments (Alcorn et al., 1991; Etges, de Oliveira, Noor, & Ritchie, 2010; Fogleman & Starmer, 1985; Havens & Etges, 2013; Starmer, 1982; Starmer, Schmedicke, & Lachance, 2003). Cacti were fermented for 1 week, with the exception of organ pipe cactus that was fermented for 5 weeks. The choice of fermentation time was based on work by Date et al. (2013). In this study, the attraction of each fly population to different fermentation stages of their respective host cacti was determined and two‐choice tests revealed that, unlike other cacti, organ pipe cactus was most attractive to flies after 5 weeks of fermentation. For experiments examining the influence of microorganism and cactus substrate on volatile composition, 10 g of each cactus was sterilized and placed into a sterile glass vial. Each sample was inoculated with an individual yeast or bacterium species at equal cell count and incubated for 1 week. Three individual replicates in separate glass vials were inoculated for each cactus–microbe pairing. All cactus tissue was incubated at 30°C.

### 2.3 Host preference assay

Behavioral tests were conducted using an olfactory trap assay system detailed in Date et al. (2013). Funnel traps were placed symmetrically within a 6 cm (H) × 15 cm (Ø) arena. Two grams of fermenting cactus was used per trap. To prevent desiccation, a cotton ball with 20 ml of water was placed into the arena. Assays were performed in the dark and the number of flies captured was recorded after 48 hr. Twenty flies were released into the testing arena with five replicate tests per sex, population, and cactus comparison. Flies were tested at 10–12 days posteclosion and starved overnight on 1% agar prior to the experiment. Each population was given a choice between all possible cactus combinations.

### 2.4 Individual microorganism fermentations and GC‐MS parameters

The volatile composition of each replicate of the cactus–microbe pairings was obtained through headspace solid‐phase microextraction (SPME). A SPME fiber (polydimethylsiloxane/divinylbenzene, Sigma‐Aldrich, St. Louis, MO) was exposed to the fermenting cactus headspace in a septum‐sealed glass vial for 1 hr. After collection, the volatiles were desorbed from the fiber in the multimode injection port of an Agilent 7890A gas chromatograph (GC) for 1 min at 250°C. Volatiles were then separated on a fused silica capillary column (50 m, 0.32 mm, 0.25 μm; Supelco Nukol, Bellefonte, PA) with a 5‐m guard column (5 m, 0.32 mm; Restek Rxi, Bellefonte, PA) with a He flow of 1.5 ml/min. The GC temperature program was 40°C for 1 min, followed by a ramp to 210°C at 7°C/min, and then a hold for 15 min. Following separation, column effluent was split (1:1) between the flame ionization detector (FID) and the mass selective detector (MSD) using a two‐way splitter (Agilent G3180B) at a constant pressure of 31 kPa. The Agilent 5975C quadrupole MSD was operated with an electron‐impact ionization of 70 eV and scanned a mass range of m/z 35–500 at 2 scans per second. Compounds were assigned using authentic standards (for 36 compounds, reported in Table S1), library databases (NIST 2008 and Wiley 2009), published spectra, spectral interpretation, and retention times. For library database identification, we used the Probability Based Matching (PBM) algorithm (Agilent Chem Station) and the NIST MS Search Program. Kovats retention indices were assigned to all compounds using a suite of *n*‐alkanes from C_7_ to C_40_ (Supelco) and are reported in Table S1. Compounds were assigned manually using the PBM and NIST programs by visual verification of the sample and the library spectra. Overall, all compounds scored above 80% in the PBM and/or 60% in the NIST with average match values of 88.4% and 63.6%, respectively. Identity match percentages were similar to those of standards. Relative abundances were determined from FID peak areas as previously established for volatile compounds of varying compound classes (Elke et al., 1999; Menetrez & Foarde, 2002; Zhang & Li, 2010).

### 2.5 Rearing flies on cactus rots

Empty 8‐dram glass vials with 5 g of gravel were autoclaved and 15 g aliquots of fermented cactus were added to each vial. All four cacti were fermented as described above. To control for larval density, flies for each of the four populations were allowed to lay eggs on cactus–banana–agar medium in egg collection chambers for 12 hr. Fifty first‐instar larvae were then hand‐picked within 12 hr of emergence and placed onto the fermenting cactus tissue in each vial. Three replicate vials of 50 larvae each were set up per population and cactus. The experiment was conducted at 25°C, under a 12‐hr L/D cycle.

### 2.6 Life‐history trait measurements

For each vial, the number of flies that emerged was recorded every 12 hr. Development time (DT) was calculated as the time from the placement of first‐instar larvae onto cactus tissue to adult eclosion. Total viability was measured as the proportion of emerging adults relative to the number of larvae seeded in each vial. Pupal viability was measured as the proportion of total pupae relative to the number of larvae seeded in each vial. On emergence, flies were equally divided into two groups and preserved in 70% ethanol or frozen at −20°C for measurements of thorax length or dry body weight, respectively. Thorax length was measured as the distance from the anterior margin of the thorax to the posterior tip of the scutellum, using an ocular micrometer. For adult dry body weight measurements, the flies were baked at 60°C for 24 hr and then individually weighed on a microbalance. DT, thorax length, and body weight measurements were scored in both sexes separately.

### 2.7 Statistical analyses

The behavioral data was analyzed using paired *t* tests and the *p*‐values were corrected for multiple testing with a false discovery rate of <0.05 (Benjamini & Hochberg, 1995). Statistical analyses for variation in DT, thorax length, and body weight were conducted using ANOVA model *Y = *μ *+ P + S + C + P X S + P X C + S X C + P X S X C + E*, where μ is the overall mean, population (*P*), sex (*S*)*,* and cactus treatment (*C*) were the fixed effects, and *E* is the within‐vial variance. To assess how each population varies on the different hosts, we subsequently performed separate population‐specific ANOVAs for sex, and treatment according to the model *Y = *μ *+ S + C + S X C + E*. Variance in total and pupal viability was analyzed in the same way except that sex was not considered as a factor. For populations in which the S X C interaction was significant, a separate sex‐specific ANOVA was performed. Mojave population flies reared on organ pipe cactus were excluded from the population‐ and sex‐specific ANOVAs due to low survival of flies on this substrate. Each ANOVA was followed by a Tukey–Kramer post hoc test. Additionally, relative performance indices (RPI) were calculated for each population–cactus pairing using a modified version of the equation found in Krebs and Barker (1993): RPI = (percent viability × body weight)/(development time). All analyses were conducted using JMP Pro 12 software (SAS Institute, Cary, NC).

## 3 Results

### 3.1 Olfactory preferences vary depending on population and substrate

**Figure 1:**
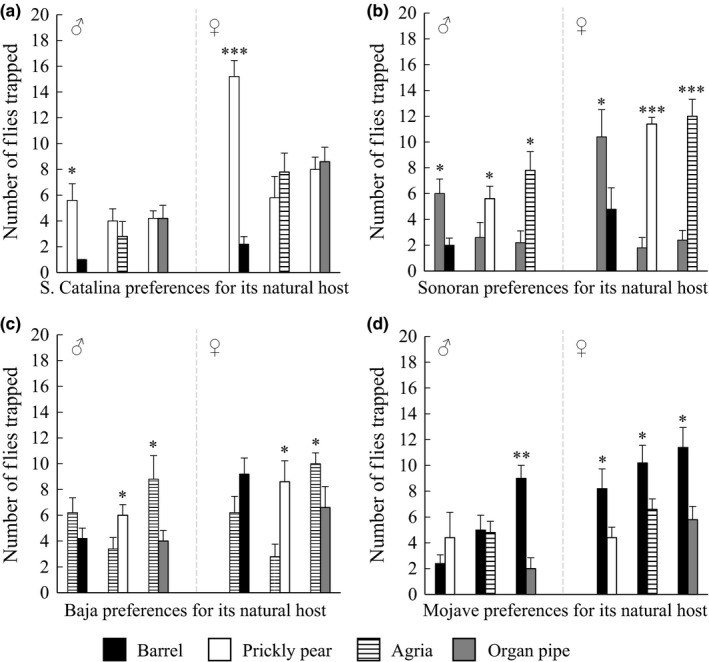
Host preference behavior of each population for its host cactus relative to an alternative cacti using two‐choice assays. Preferences of the (a) S. Catalina, (b) mainland Sonoran, (c) Baja, and (d) Mojave populations for their respective host plants. Behavioral preferences are shown by sex as mean ± standard error, and statistical significance is depicted by asterisks (*p <* *.05; **p <* *.01; ***p <* *.001)

Given that the volatiles emitted during fermentation are used by *D. mojavensis* for appropriate host plant identification, we tested the hypothesis that flies show a stronger preference for their own host cactus over alternative cacti. Flies from each of the four *D. mojavensis* populations were given a choice between two cacti inoculated with yeast and bacteria commonly found on fermenting cactus (Alcorn et al., 1991; Fogleman & Heed, 1989; Fogleman & Starmer, 1985; Starmer, 1982; Starmer et al., 2003). The number of flies trapped in these two‐choice assays was recorded and significant differences in olfactory preference among populations were found that depend on cactus substrate choice. Both sexes of the S. Catalina and mainland Sonoran populations showed a significant preference for their hosts (prickly pear and organ pipe, respectively) over barrel cactus (Figure 1a,b; Table S2a). However, this host‐specific preference was not observed in tests with the other cacti that are not their natural host: The S. Catalina population showed no preference and the mainland Sonoran population preferred alternative substrates (prickly pear and agria). Differences in preference were also observed for the Baja population that was preferentially attracted to its host, agria, over organ pipe cactus, but showed either no preference (barrel) or attraction (prickly pear) to the remaining alternate cacti (Figure 1c). Finally, most notable were the behavioral responses of the Mojave population, which uses barrel cactus. Females consistently showed a significant preference for barrel cactus over all other alternative cacti (Figure 1d). Males also showed a preference for barrel relative to organ pipe cactus, but males were equally attracted to the remaining alternatives.

**Figure 2:**
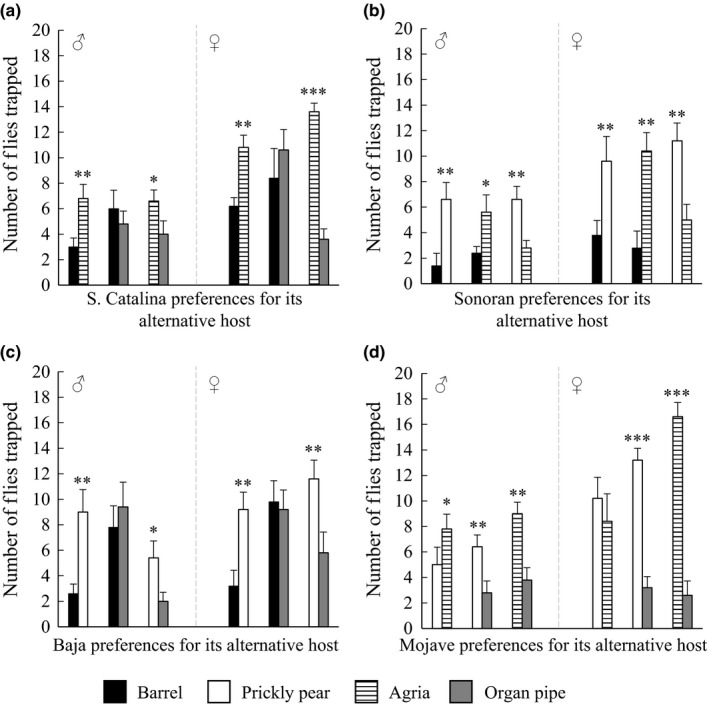
Host preference behavior for alternative hosts for each population using two‐choice assays. Preferences of males and females of the (a) S. Catalina, (b) mainland Sonoran, (c) Baja, and (d) Mojave populations. Behavioral preferences are shown by sex as mean ± standard error, and statistical significance is depicted by asterisks (*p <* *.05; **p <* *.01; ***p <* *.001)

To further examine preferences among populations, we also performed two‐choice tests between all possible combinations of alternative cacti for each population. Based on these two‐choice tests, we inferred an ordered preference hierarchy among the three alternative cacti for each population. Future multichoice tests, however, will be needed to further test these hierarchies. For the S. Catalina and mainland Sonoran populations, the preference hierarchies for alternate cacti were as follows: (a) agria > barrel and organ pipe cactus and (b) prickly pear > agria > barrel cactus, respectively (Figure 2a,b; Table S2b). For the Baja population, fly preferences for prickly pear were greater than those for either barrel or organ pipe cactus (Figure 2c). Finally, in the Mojave population, there was a reduced preference for organ pipe cactus (i.e., agria and prickly pear > organ pipe; Figure 2d). In short, our results show that the four populations of *D. mojavensis* vary in the degree of preference for their host plant in two‐choice tests, and in the case of females from the Mojave population, the preference for its host plant is highly pronounced. Moreover, when given choices of only the alternatives, preferences for barrel and organ pipe cactus were lower overall across the remaining three populations.

### 3.2 Volatile composition of cactus substrates varies with microorganism and substrate

**Figure 3:**
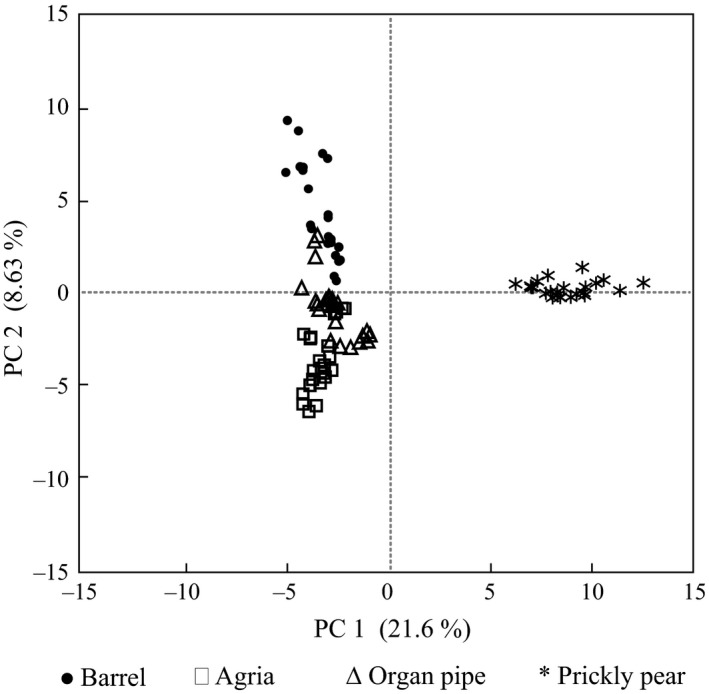
Principal component analysis of volatile compounds from all four different cacti as a result of single inoculation with eight different microorganisms

**Figure 4:**
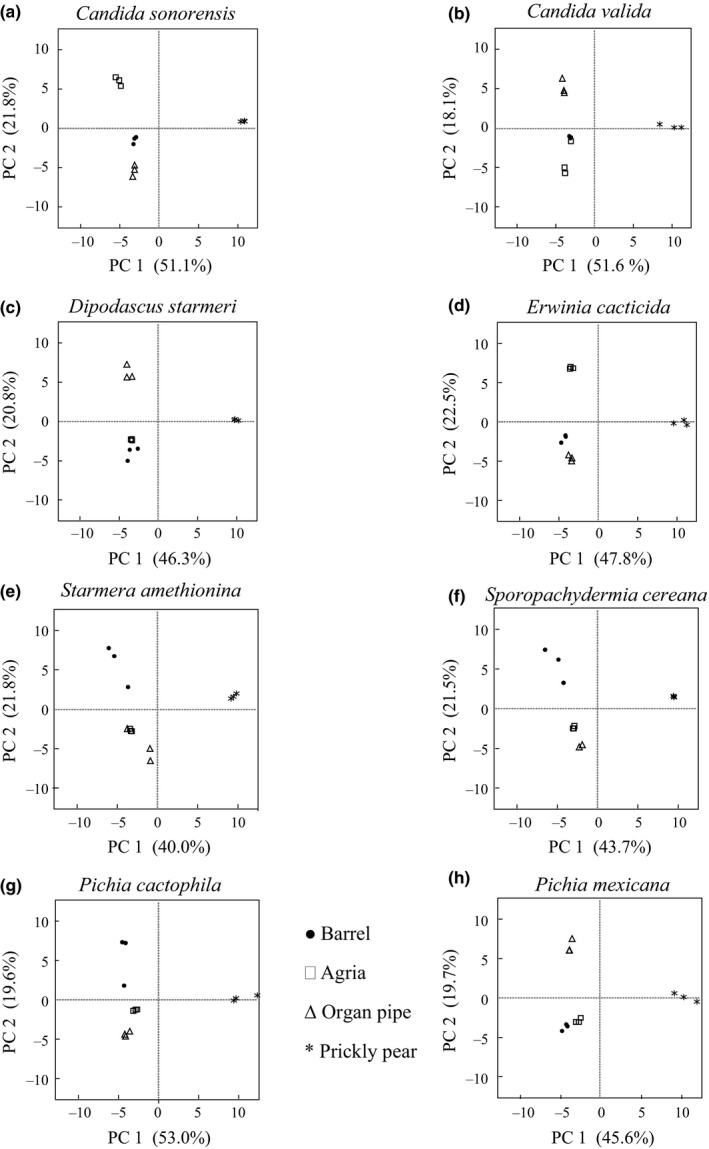
Individual principal component analyses of volatile compounds from all four cacti after individual inoculations with eight different microorganisms (a) *Candida sonorensis,* (b) *Candida valida,* (c) *Dipodascus starmeri,* (d) *Erwinia cacticida,* (e) *Starmera amethionina,* (f) *Sporopachydermia cereana,* (g) *Pichia cactophila,* and (h) *Pichia mexicana*

We observed significant population differences in olfactory preferences that depend on cactus substrate. To further elucidate factors underlying the observed preferences, we examined the relationship between the plant, microorganism, and volatile profile produced. Each cactus was inoculated with an individual yeast or bacterium previously used in the preference tests. The volatiles emitted following fermentation were then evaluated by GC‐MS. One hundred and thirty volatile compounds were detected that differed in their relative amounts between cacti and several interesting results emerged. First, principal component analysis (PCA) using this data set revealed substrate‐specific differences (Figure 3; Table S3). Irrespective of the microorganism, the agria and organ pipe samples generally overlapped, suggesting that their volatile headspaces are fairly similar. This is perhaps not unexpected given that these two cacti belong to the same genus (*Stenocereus*). Individual samples of barrel cactus and prickly pear formed distinct clusters, particularly in the case of the latter. Second, PCA performed for each individual microorganism revealed differences among cacti in the relative similarities of their host plant volatile compositions (Figure 4). Principal component (PC) 1 accounted for 40%–53% of the variability in the data and PC2 from 18.1%–22.5%. (Tables S4–11). Across all microbial treatments, replicate prickly pear samples formed a distinct cluster relative to the other cactus substrates. Clustering of prickly pear was influenced by a group of compounds (benzyl alcohol, methyl salicylate, linalool oxide, linalool, prenol, and perillene; Table S3–11) consistently seen among the highest scoring eigenvectors along PC1 (Figure 4) in all inoculations. Moreover, inoculation with a subset of yeasts, *Starmera amethionina*,* Sporopachydermia cereana,* and *Pichia cactophila*, resulted in distinct clustering of barrel samples. Inoculation with these yeasts also resulted in fairly similar agria and organ pipe volatile profiles. However, for the five remaining microorganisms, the pattern of similarity among the volatile compositions of different host plants varied. Our analyses suggest that the substrate, microorganism, and substrate‐by‐microorganism interactions together contribute to volatile headspace composition.

### 3.3 Population differences were observed in fly viability, developmental time, and body size and depend on cactus rearing substrate

**Figure 5:**
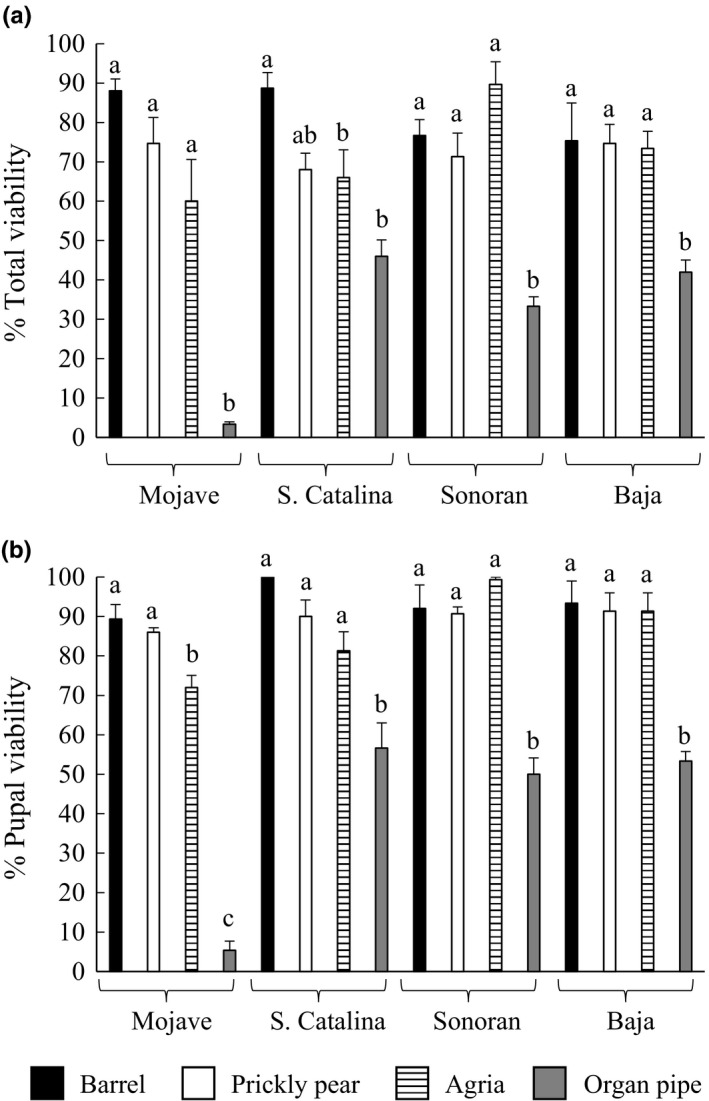
Population differences in viability of flies reared on different cacti. Measurements were obtained for each population reared on the four different host plants for (a) total viability and (b) pupal viability. Trait measurements (mean ± standard error) are shown. Significant differences within a population were determined using a Tukey–Kramer post hoc test and denoted by different letters

Adult host preference behavior can have direct consequences on offspring performance (Gripenberg, Mayhew, Parnell, & Roslin, 2010). To evaluate the effects of host plant substrate on fly performance, we measured viability, DT, and body size for each of the four populations reared independently on all four cacti. For all traits, we observed an effect of rearing substrate. Total viability was significantly reduced when populations were reared on organ pipe cactus, with the Mojave population showing the greatest reduction (Figure 5a; Table S12a). A similar pattern of reduced viability on organ pipe cactus was also found for pupal viability, with the Mojave population additionally showing slightly reduced viability on agria both of which are not its host plant (Figure 5b; Table S12b).

**Figure 6:**
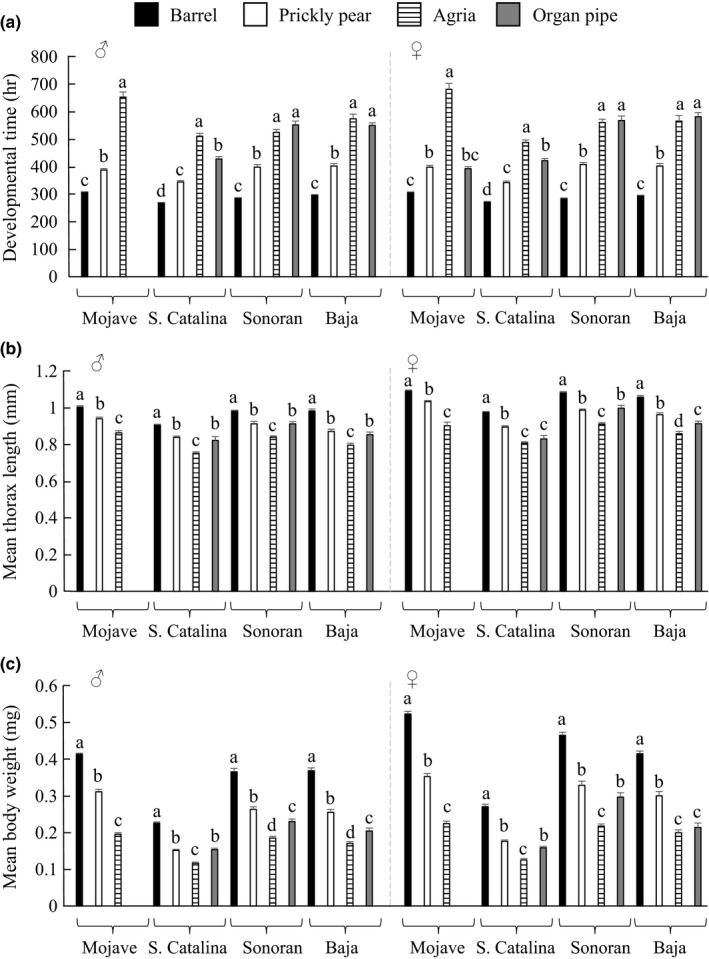
Population difference in development time and adult body size when larvae were reared on different cacti. Measurements for each population are as follows: (a) development time, (b) thorax length, and (c) body weight. Trait measurements (mean ± standard error) are shown by sex. Significant differences within a population were determined using a Tukey–Kramer post hoc test and denoted by different letters. With the exception of female development time measurements, Mojave population flies reared on organ pipe cactus were excluded from sex‐specific ANOVAs due to low survival of the flies on this substrate

In terms of DT and body size, flies reared on barrel cactus had the shortest DT for all populations, followed by prickly pear (Figure 6a; Table S12c). The longest DT overall was observed when flies were reared on agria and organ pipe cactus. The Mojave population in particular showed a marked lack of development on organ pipe cactus, particularly in males, in which only one fly eclosed. Finally, we examined the effect of cactus substrate on two measures of body size: thorax length and dry body weight for each population (Figure 6b,c; Table S12d,e). Thorax length differed significantly between sexes, populations, and by rearing substrate. Females were larger than males, consistent with many insect species, including *Drosophila* (Stillwell, Blanckenhorn, Teder, Davidowitz, & Fox, 2010). Moreover, flies from the four populations differed in their length, irrespective of rearing substrate. The Mojave population had the greatest thorax length followed by the mainland Sonoran, Baja, and S. Catalina flies, respectively. Also, thorax length varied with rearing substrate. Rearing of flies on barrel cactus resulted in increased thorax length in all populations. Development on prickly pear and organ pipe yielded flies of similar length, and agria produced the shortest flies. For a second measure of body size, dry body weight, the pattern of effects was similar.

## 4 Discussion

Populations varied in their olfactory preferences for different fermented substrates. In previous studies of preference for synthetic mixtures or artificially fermented agria versus organ pipe cactus, populations tended to retain the ancestral preference for agria (Fellows & Heed, 1972; Newby & Etges, 1998). This observation was also seen in our study. Additionally, our extension of such comparisons to all combinations of cacti and populations revealed further trends. First, preference for organ pipe cactus was generally reduced relative to all other cacti. Both agria and organ pipe contain triterpene glycosides and lipids that can have detrimental effects on *Drosophila* viability (Fogleman & Heed, 1989; Kircher, 1977; Starmer & Fogleman, 1986). However, these cacti differ in the proportion and composition of these compound classes, with organ pipe being the poorer breeding substrate (Kircher, 1982; Etges & Heed, 1987; Fogleman & Armstrong 1989). Second, Mojave population females, in particular, had marked preferences for their own host plant, barrel cactus, when given a choice of it versus an alternative. This result is consistent with past research, suggesting that the Mojave population diverged with host shift in its genetic structure, olfactory electrophysiological and behavioral responses to cactus volatiles (Ross & Markow, 2006; Date et al., 2013; Crowley‐Gall et al., 2016). Finally, it is interesting that the mainland Sonoran population prefers its host, organ pipe cactus, but only when paired with barrel. The mainland Sonoran flies used in this study were collected at Organ Pipe National Monument, a location where both organ pipe and barrel cactus are found (Schmidt et al., 2007). Future work is needed to assess geographic variation in olfactory preference and its potential association with plant distribution patterns.

Understanding the determinants of insect host preference behavior involves teasing apart the significance of the host plant and microbial community to the volatile cues mediating insect attraction. Previous work in *D. melanogaster* has suggested that the role of yeast is underappreciated despite its importance to host plant identification, discrimination, and, potentially, divergence among populations. Specifically, it has been proposed that the chemical signal emitted from the plant substrate alone is less significant to olfactory behavioral responses than that of the yeast‐produced volatile fermentation products (Becher et al., 2012). Our previous work in the *D. mojavensis* system supports this observation to the extent that the cacti, in the absence of fermentation by microorganisms, elicit only modest fly attraction (Date et al., 2013). However, this study illustrates the complex interplay between microorganism and substrate and the importance of their interactions in mediating differences in volatile composition. Microorganism–cactus interactions result in distinct volatile profiles (this study; Date et al., 2013). In nature, the frequency of yeast species on different host plants varies (Fogleman, Starmer, & Heed, 1981). In a study of yeasts isolated from rots of three of the four *D. mojavensis* host plants (prickly pear not studied), Fogleman et al. (1981) consistently found *P. cactophila* and *C. sonorensis* on all substrates. *Pichia mexicana*, however, was absent from barrel rots. Also, the phyletic division of cacti has been shown to impact yeast communities (Starmer, 1980; Starmer & Fogleman, 1986; Starmer, Kircher, & Phaff, 1980). Agria and organ pipe belong to the same *Stenocereus* genus and we hypothesized that their headspaces would be quite similar, and this was demonstrated by the PCA of all samples regardless of the type of inoculation. However, distinct headspaces for these two cacti were observed when the substrates were inoculated with a subset of individual microorganisms. These results support the importance of both the microbial community and plant substrate in insect host preference and set the stage for future studies addressing how the native microbe community affects the volatile composition of cactus rots and insect behavior in the field.

Adult host preference can affect offspring performance. Several studies in cactophilic drosophilids have shown clear associations between host plant preference and performance. For example, sister species *D. buzzatii* and *D. koepferae*, which prefer prickly pear and columnar cactus, respectively, show a reduced performance in multiple life‐history traits when reared on or exposed to a nonhost cacti (e.g., Fanara, Fontdevila, & Hasson, 1999; Hurtado, Soto, Orellana, & Hasson, 2012; Soto, Goenaga, Hurtado, & Hasson, 2012). The effects of rearing substrate on the evolution of life‐history traits and performance in the *D. mojavensis* system have also been extensively studied for the Baja and mainland Sonoran populations and their respective host cacti. These studies revealed that rearing substrate influences life‐history traits including egg–adult viability, DT, body size, epicuticular hydrocarbons profiles as well as expression of genes associated with metabolism and detoxification (e.g., Etges, 1993; Etges & de Oliveira, 2014; Etges et al., 2010; Havens & Etges, 2013; Matzkin, Watts, Bitler, Machado, & Markow, 2006). In our study, we extended the assessment of rearing substrate effects on performance to include all the populations and host cacti. First, consistent with previous work, the Baja flies were smaller in thorax length than the mainland Sonoran flies regardless of whether they were reared on agria or organ pipe cactus (Etges, 1989, 1993). This difference in thorax size has been suggested to impact dispersal rates among populations (Etges, 1993). Second, in a study of host cactus effects on fitness in the Baja and mainland Sonoran populations, Etges and Heed (1987) noted a reduction in egg–adult viability when flies were reared on organ pipe cactus. We observed a more marked reduction in our measure of larva–adult viability on organ pipe, consistent with it being a poorer breeding substrate (Etges & Heed, 1987; Fogleman & Armstrong 1989). Etges and Heed (1987) also found a shorter egg–adult DT for the Baja population. We did not, however, observe a major reduction in larvae–adult DT between the Baja and mainland Sonoran populations. Several factors may account for the difference among studies. Larval density has been shown to influence DT and viability, with the most notable differences at increased density (Etges & Heed, 1987). The studies also differ in their measures of viability and DT, and experimental differences exist in the stage of cactus fermentation used. Our study examined the effects of 1 week fermented cacti on *D. mojavensis*, with the exception of organ pipe cactus that was fermented for 5 weeks. The increased fermentation period for organ pipe cactus was selected based on Date et al. (2013). In preference experiments to identify the fermentation stage(s) attractive to flies, organ pipe cactus was attractive at a later stage than that observed for other cacti. Finally, dissimilarities among studies in these life‐history traits have also been suggested to be due to differences in tissue quality (Etges, 1989, 1993). In summary, our results show differences among populations in larval performance (DT) and that larva develop faster on barrel and prickly pear cacti with prolonged DT on agria and organ pipe. Thus, the cactus rearing environment had an effect on DT, but unlike studies in other cactophilic *Drosophila* species, in this system performance was not clearly associated with host plant (Table S13).

Research on host attraction in *Drosophila* has focused on identifying the volatiles produced by yeasts on a given substrate (Becher et al., 2012; Scheidler, Liu, Hamby, Zalom, & Syed, 2015). These studies have been influential in identifying key yeast volatile compounds that can drive attraction in yeast feeding insects. However, understanding how the microorganism–plant interaction influences volatile composition and insect preference continues to be a challenge. This study illustrates the complexity of insect–microorganism–plant relationships and the importance of considering these factors and their associations on insect preference and performance.

## Author contributions

All authors conceived the ideas and designed methodology; PD and ACG collected the data; PD, ACG, and AFD analyzed the data; PD, ACG, and SMR led the writing of the manuscript. All authors contributed critically to the drafts and gave final approval for publication.

## Data accessibility

All data used in this manuscript are present in the manuscript and its supporting information.

## Conflict of interest

None declared.
