# Supplementary material for: Population differences in host plant preference and the importance of yeast and plant substrate to volatile composition
Source: Ecol Evol. 2017 Apr 18;7(11):3815–25. doi: 10.1002/ece3.2993 (PMC5468138; doi:10.1002/ece3.2993)
Supplement: Supplementary file 1 [file ECE3-7-3815-s001.docx]

**SUPPORTING INFORMATION**

**Table S1.** Retention times, Kovats retention indices, and methods of compound identification for GC-MS.

|  | **Compound Name** | **Retention Time** | **Kovats Indices** | **Identification^1^** |
| --- | --- | --- | --- | --- |
| 1 | Octane | 5.344 | 867 | L |
| 2 | Acetone | 5.584 | 882 | L |
| 3 | Isopropyl alcohol | 6.557 | 942 | L, S |
| 4 | 2-Butanol | 7.879 | 1026 | L |
| 5 | Ethyl butanoate | 7.998 | 1034 | L, S |
| 6 | Ethyl 2-methylbutanoate | 8.244 | 1050 | L |
| 7 | Methyl thioacetate | 8.273 | 1052 | L |
| 8 | Ethyl isovalerate | 8.503 | 1067 | L |
| 9 | Dimethyl disulfide | 8.712 | 1081 | L |
| 10 | Hexanal | 8.855 | 1091 | L |
| 11 | Ethyl carbonate | 9.147 | 1109 | L |
| 12 | 3-Pentanol | 9.272 | 1116 | L, Sp |
| 13 | Isoamyl acetate/Pentyl isobutyrate^2^ | 9.41 | 1124 | L, S |
| 14 | 2-Pentanol | 9.505 | 1129 | L |
| 15 | Butanoic acid, 1-methylpropyl ester | 9.524 | 1130 | L |
| 16 | Isobutyl butanoate | 10.096 | 1163 | L |
| 17 | 4-Methyl-2-pentanol | 10.296 | 1174 | L |
| 18 | Pentyl acetate | 10.378 | 1179 | L, S |
| 19 | 2-Heptanone | 10.644 | 1194 | L, S |
| 20 | Isoamyl propionate | 10.678 | 1196 | L, S, Sp |
| 21 | 3-Hexanol | 10.863 | 1206 | L |
| 22 | Isoamyl alcohol/2-methyl-1-butanol^2^ | 11.076 | 1218 | L |
| 23 | 2-Penten-1-ol, acetate, (Z)- | 11.338 | 1232 | L |
| 24 | 2-Pentylfuran | 11.418 | 1236 | L, S |
| 25 | Ethyl caproate | 11.432 | 1237 | L, S |
| 26 | 6-Methyl-2-heptanone | 11.662 | 1249 | L |
| 27 | Prenyl acetate | 11.899 | 1262 | L, S |
| 28 | 1-Pentanol | 11.934 | 1264 | L |
| 29 | Isobutenylcarbinol | 11.972 | 1266 | L |
| 30 | 3-Octanone | 12.039 | 1270 | L, S |
| 31 | Isoamyl butyrate | 12.088 | 1273 | L, S |
| 32 | Ethenylbenzene | 12.104 | 1273 | L |
| 33 | 2-Propenylbenzene | 12.211 | 1279 | L |
| 34 | Hexyl acetate | 12.258 | 1282 | L, S |
| 35 | Isoamyl-2-methyl butyrate | 12.317 | 1285 | L |
| 36 | 5-Methyl-2-hexanol | 12.385 | 1289 | L |
| 37 | 2-Octanone | 12.599 | 1300 | L |
| 38 | Isoamyl isovalerate | 12.627 | 1302 | L |
| 39 | Trans-2-(2-Pentenyl)furan | 12.765 | 1309 | L |
| 40 | Acetoin | 12.877 | 1315 | L, S |
| 41 | Hexanenitrile | 12.93 | 1318 | L |
| 42 | 2-Heptanol | 13.161 | 1331 | L |
| 43 | Prenol | 13.325 | 1340 | L |
| 44 | 2-Heptenal | 13.471 | 1348 | L |
| 45 | 4-Pentenyl butyrate | 13.506 | 1350 | L |
| 46 | 6-Methyl-5-hepten-2-one | 13.601 | 1355 | L, S |
| 47 | 3-methylcyclopentanol | 13.702 | 1360 | L |
| 48 | 1-Hexanol | 13.847 | 1368 | L, S |
| 49 | 2-Isopropyl pyrazine | 14.026 | 1378 | L |
| 50 | 6-Methyl-2-heptanol | 14.123 | 1383 | L |
| 51 | Heptyl acetate | 14.125 | 1384 | L |
| 52 | Methyl octanoate | 14.419 | 1400 | L |
| 53 | 3-Hexen-1-ol, (Z)- | 14.455 | 1402 | L |
| 54 | 3-Octanol | 14.478 | 1403 | L |
| 55 | 2-Nonanone | 14.522 | 1405 | L, S |
| 56 | Nonanal | 14.585 | 1409 | L, S |
| 57 | Dimethyl trisulfide | 14.587 | 1409 | L, S |
| 58 | 2-Methyl-3-isopropylpyrazine | 14.693 | 1415 | L, Sp |
| 59 | 1-Nitropentane | 14.718 | 1416 | L |
| 60 | 2-Hexen-1-ol | 14.826 | 1423 | L |
| 61 | Hexyl butanoate | 14.886 | 1426 | L |
| 62 | Perillene | 14.933 | 1429 | L |
| 63 | Benzene,m-di-tert-butyl | 15.057 | 1436 | L |
| 64 | Ethyl octanoate | 15.205 | 1444 | L, S |
| 65 | 2-Methoxy-3-(methylethyl)-pyrazine | 15.297 | 1449 | L |
| 66 | 2-Octenal | 15.404 | 1455 | L |
| 67 | 1-Octen-3-ol | 15.493 | 1460 | L, S |
| 68 | Linalool oxide | 15.583 | 1465 | L |
| 69 | 1-Heptanol | 15.682 | 1471 | L |
| 70 | 6-Methyl-5-hepten-2-ol | 15.77 | 1476 | L |
| 71 | Octyl acetate | 15.928 | 1485 | L |
| 72 | 2-Ethyl-1-hexanol | 16.233 | 1502 | L, S |
| 73 | Copaene | 16.397 | 1512 | L |
| 74 | 2,5-Bis(1-methylethyl)pyrazine | 16.406 | 1512 | L, Sp |
| 75 | Methyl 2-hydroxy-3-methyl pentanoate | 16.467 | 1516 | L, Sp |
| 76 | 1-Nitrohexane | 16.572 | 1522 | L |
| 77 | 2,4-Heptadienal | 16.643 | 1526 | L |
| 78 | 2-Nonanol | 16.713 | 1531 | L, S |
| 79 | 3,3-Dimethylcyclohexanol | 16.789 | 1535 | L |
| 80 | 2-Isobutyl-3-methoxypyrazine | 16.968 | 1546 | L |
| 81 | Linalool | 17.171 | 1558 | L, S |
| 82 | Benzaldehyde | 17.225 | 1561 | L |
| 83 | Dihydro-2-methyl-3(2H)-thiophenone | 17.313 | 1566 | L |
| 84 | 1-Octanol | 17.445 | 1574 | L |
| 85 | Nonyl acetate | 17.704 | 1589 | L |
| 86 | Acetic acid | 17.825 | 1596 | L |
| 87 | Methyl decanoate | 17.996 | 1607 | L |
| 88 | 2-Undecanone | 18.18 | 1618 | L |
| 89 | 6-Methyl-3,5-heptadien-2-one | 18.368 | 1630 | L |
| 90 | 4-Terpineol | 18.391 | 1631 | L |
| 91 | Benzonitrile | 18.663 | 1648 | L |
| 92 | Methyl benzoate | 18.838 | 1659 | L, S |
| 93 | 3-(Methylthio)propyl acetate | 18.861 | 1660 | L |
| 94 | 1-Nonanol | 19.135 | 1677 | L |
| 95 | Isopropyl benzoate | 19.274 | 1686 | L, S |
| 96 | Acetophenone | 19.431 | 1695 | L, S |
| 97 | Ethyl benzoate | 19.541 | 1702 | L, S |
| 98 | Disulfide, methyl (methylthio)methyl | 19.547 | 1703 | L |
| 99 | Butanoic acid | 19.763 | 1717 | L |
| 100 | Ionene | 19.864 | 1723 | L |
| 101 | Pentanoic acid | 20.249 | 1748 | L |
| 102 | 3-(Methylthio)-1-propanol | 20.366 | 1756 | L |
| 103 | Benzyl acetate | 20.462 | 1762 | L |
| 104 | sec-Butyl benzoate | 20.805 | 1784 | L |
| 105 | Isopropyl phenylacetate | 20.85 | 1787 | L |
| 106 | Propyl benzoate | 20.952 | 1794 | L, S |
| 107 | Methyl phenylacetate | 20.985 | 1796 | L |
| 108 | Ethyl phenylacetate | 21.335 | 1819 | L, Sp |
| 109 | Methyl salicylate | 21.389 | 1823 | L, S |
| 110 | 2-Tridecanone | 21.499 | 1831 | L |
| 111 | Phenethyl acetate | 21.82 | 1852 | L, S |
| 112 | Ethyl salicylate | 21.86 | 1855 | L |
| 113 | 1-Phenyl-2-propanol | 21.983 | 1863 | L |
| 114 | Geranylacetone | 22.223 | 1880 | L |
| 115 | Guaiacol | 22.566 | 1903 | L, S |
| 116 | Benzyl alcohol | 22.818 | 1921 | L |
| 117 | Pentyl benzoate | 23.251 | 1952 | L |
| 118 | Phenylethyl alcohol | 23.358 | 1959 | L, S |
| 119 | Phenethyl butyrate | 23.951 | 2001 | L |
| 120 | Creosol | 23.959 | 2002 | L |
| 121 | 3-Methyl-3-butenyl benzoate | 24.441 | 2037 | L |
| 122 | Isoamyl phenylacetate | 24.468 | 2039 | L |
| 123 | Phenol | 24.637 | 2052 | L, S |
| 124 | 4-Ethylguaiacol | 24.98 | 2077 | L, S |
| 125 | Benzenepropanol | 25.265 | 2098 | L |
| 126 | 4-Methylphenol | 25.673 | 2117 | L, S |
| 127 | Eugenol | 26.939 | 2172 | L |
| 128 | 4-Ethylphenol | 27.03 | 2176 | L |
| 129 | 2-Methoxy-4-vinylphenol | 27.455 | 2194 | L |
| 130 | Ethyl palmitate | 27.713 | 2234 | L |

^1^Methods of compound identification: L (NIST and PBM library databases), S (authentic standard) and Sp (Spectral interpretation). ^2^Compounds co-eluting at the same time on the column.

**Table S2.** Host preference behavior experiments were analyzed using t-test statistics. Host preference behavior of each *D. mojavensis* population for (a) cacti relative to its natural host (b) alternative cacti.

| **(a)** Host preference behavior of each *D. mojavensis* population for cacti relative to its natural host | | | | | |
| --- | --- | --- | --- | --- | --- |
| **Population** | **Sex** | **Cactus comparison** | **t-ratio** | ***p-values*** | **FDR-adjusted *p-values*** |
| Mojave | M | Barrel vs Prickly pear | 0.96 | 0.1821 | 0.1510 |
| Mojave | M | Barrel vs Agria | 0.14 | 0.4460 | 0.3054 |
| Mojave | M | Barrel vs Organ pipe | -5.37 | 0.0003 | 0.0012 |
| Mojave | F | Barrel vs Prickly pear | -2.19 | 0.0298 | 0.0385 |
| Mojave | F | Barrel vs Agria | 2.27 | 0.0262 | 0.0375 |
| Mojave | F | Barrel vs Organ pipe | -3.04 | 0.0081 | 0.0142 |
| S. Catalina | M | Prickly pear vs Barrel | 3.57 | 0.0036 | 0.0113 |
| S. Catalina | M | Prickly pear vs Agria | 0.80 | 0.2229 | 0.1672 |
| S. Catalina | M | Prickly pear vs Organ pipe | 0.00 | 0.5000 | 0.3281 |
| S. Catalina | F | Prickly pear vs Barrel | 9.48 | 0.0001 | 0.0005 |
| S. Catalina | F | Prickly pear vs Agria | -0.91 | 0.1959 | 0.1543 |
| S. Catalina | F | Prickly pear vs Organ pipe | -0.41 | 0.3469 | 0.2483 |
| mainland Sonoran | M | Organ pipe vs Barrel | 3.16 | 0.0067 | 0.0142 |
| mainland Sonoran | M | Organ pipe vs Prickly pear | 1.97 | 0.0422 | 0.0443 |
| mainland Sonoran | M | Organ pipe vs Agria | -3.24 | 0.0059 | 0.0142 |
| mainland Sonoran | F | Organ pipe vs Barrel | 2.09 | 0.0352 | 0.0396 |
| mainland Sonoran | F | Organ pipe vs Prickly pear | 10.12 | 0.0001 | 0.0005 |
| mainland Sonoran | F | Organ pipe vs Agria | -6.39 | 0.0001 | 0.0005 |
| Baja | M | Agria vs Barrel | -1.42 | 0.0965 | 0.0844 |
| Baja | M | Agria vs Prickly pear | 2.15 | 0.0318 | 0.0385 |
| Baja | M | Agria vs Organ pipe | -2.39 | 0.0220 | 0.0347 |
| Baja | F | Agria vs Barrel | 1.68 | 0.0655 | 0.0607 |
| Baja | F | Agria vs Prickly pear | 3.06 | 0.0078 | 0.0142 |
| Baja | F | Agria vs Organ pipe | -1.85 | 0.0504 | 0.0496 |

| **(b)** Host preference behavior of each *D. mojavensis* population for alternative host cacti. | | | | | |
| --- | --- | --- | --- | --- | --- |
| **Population** | **Sex** | **Cactus comparison** | **t-ratio** | ***p-values*** | **FDR-adjusted *p-values*** |
| Mojave | M | Prickly pear vs Agria | -1.56 | 0.0792 | 0.0306 |
| Mojave | M | Prickly pear vs Organ pipe | 2.76 | 0.0123 | 0.0070 |
| Mojave | M | Agria vs Organ pipe | -3.94 | 0.0021 | 0.0028 |
| Mojave | F | Prickly pear vs Agria | 0.66 | 0.2634 | 0.0880 |
| Mojave | F | Prickly pear vs Organ pipe | 7.96 | 0.0001 | 0.0002 |
| Mojave | F | Agria vs Organ pipe | -8.82 | 0.0001 | 0.0002 |
| S. Catalina | M | Barrel vs Agria | -2.88 | 0.0102 | 0.0062 |
| S. Catalina | M | Barrel vs Organ pipe | -0.68 | 0.2587 | 0.0880 |
| S. Catalina | M | Agria vs Organ pipe | -1.91 | 0.0465 | 0.0190 |
| S. Catalina | F | Barrel vs Agria | -3.92 | 0.0022 | 0.0028 |
| S. Catalina | F | Barrel vs Organ pipe | 0.78 | 0.2284 | 0.0839 |
| S. Catalina | F | Agria vs Organ pipe | -9.45 | 0.0001 | 0.0002 |
| mainland Sonoran | M | Barrel vs Prickly pear | 3.15 | 0.0068 | 0.0045 |
| mainland Sonoran | M | Barrel vs Agria | -2.2 | 0.0296 | 0.0128 |
| mainland Sonoran | M | Prickly pear vs Agria | 3.21 | 0.0062 | 0.0045 |
| mainland Sonoran | F | Barrel vs Prickly pear | 2.57 | 0.0166 | 0.0081 |
| mainland Sonoran | F | Barrel vs Agria | -3.9 | 0.0023 | 0.0028 |
| mainland Sonoran | F | Prickly pear vs Agria | 3.34 | 0.0051 | 0.0045 |
| Baja | M | Barrel vs Prickly pear | 3.35 | 0.0051 | 0.0045 |
| Baja | M | Barrel vs Organ pipe | 0.62 | 0.2754 | 0.0880 |
| Baja | M | Prickly pear vs Organ pipe | 2.26 | 0.0268 | 0.0123 |
| Baja | F | Barrel vs Prickly pear | 3.26 | 0.0057 | 0.0045 |
| Baja | F | Barrel vs Organ pipe | -0.27 | 0.3984 | 0.1220 |
| Baja | F | Prickly pear vs Organ pipe | 2.65 | 0.0147 | 0.0077 |

**Table S3.** Principal component values for volatile compounds from the four cacti independently inoculated with eight different microorganisms^1^.

|  |  | **PC 1** | **PC 2** | **PC3** | **PC 4** |
| --- | --- | --- | --- | --- | --- |
|  | **Eigenvalue** | 28.04 | 11.22 | 9.58 | 8.39 |
|  | **Percent variation** | 21.57 | 8.63 | 7.37 | 6.45 |
|  | **Eigenvectors** |  |  |  |  |
| 1 | Octane | -0.03988 | -0.06974 | 0.02056 | 0.04565 |
| 2 | Acetone | -0.02502 | -0.01575 | -0.09962 | -0.14283 |
| 3 | Isopropyl alcohol | 0.17017 | -0.00671 | -0.03009 | -0.01682 |
| 4 | 2-Butanol | 0.13908 | 0.00172 | -0.00480 | 0.01685 |
| 5 | Ethyl butanoate | 0.10656 | 0.01096 | 0.01985 | 0.00886 |
| 6 | Ethyl 2-methylbutanoate | 0.08894 | -0.00085 | -0.01721 | 0.00835 |
| 7 | Methyl thioacetate | -0.02255 | 0.07298 | 0.04750 | -0.02732 |
| 8 | Ethyl isovalerate | 0.12898 | 0.00214 | -0.01135 | 0.01817 |
| 9 | Dimethyl disulfide | -0.03998 | 0.11902 | 0.11842 | -0.07435 |
| 10 | Hexanal | -0.03066 | 0.12804 | -0.07000 | 0.20873 |
| 11 | Ethyl carbonate | -0.02577 | -0.03512 | -0.05631 | -0.05832 |
| 12 | 3-Pentanol | -0.02444 | -0.09932 | 0.07680 | 0.07667 |
| 13 | Isoamyl acetate/Pentyl isobutyrate^2^ | 0.01784 | 0.14559 | 0.14909 | -0.13258 |
| 14 | 2-Pentanol | -0.03024 | -0.11123 | 0.06635 | 0.07043 |
| 15 | Butanoic acid, 1-methylpropyl ester | 0.07901 | -0.00283 | -0.00124 | 0.00288 |
| 16 | Isobutyl butanoate | 0.09664 | 0.00139 | 0.01646 | 0.00215 |
| 17 | 4-Methyl-2-pentanol | -0.05267 | -0.18040 | 0.13754 | 0.11565 |
| 18 | Pentyl acetate | -0.02207 | 0.12653 | 0.16699 | -0.15693 |
| 19 | 2-Heptanone | -0.06905 | -0.01359 | -0.18821 | -0.08811 |
| 20 | Isoamyl propionate | 0.08694 | 0.00254 | -0.00281 | 0.01050 |
| 21 | 3-Hexanol | -0.04014 | -0.14792 | 0.11474 | 0.09980 |
| 22 | Isoamyl alcohol/2-methyl-1-butanol^2^ | 0.15973 | 0.03785 | 0.00247 | -0.02112 |
| 23 | 2-Penten-1-ol, acetate, (Z)- | -0.01560 | 0.09685 | 0.15456 | -0.14581 |
| 24 | 2-Pentylfuran | -0.09241 | 0.16928 | 0.06028 | 0.12221 |
| 25 | Ethyl caproate | -0.00472 | -0.03347 | 0.01035 | 0.01005 |
| 26 | 6-Methyl-2-heptanone | -0.06208 | -0.04868 | -0.18645 | -0.08033 |
| 27 | Prenyl acetate | 0.09122 | 0.00256 | 0.02868 | 0.00363 |
| 28 | 1-Pentanol | -0.02498 | 0.05511 | -0.13069 | 0.10201 |
| 29 | Isobutenylcarbinol | 0.14480 | -0.02644 | -0.07989 | -0.03611 |
| 30 | 3-Octanone | -0.05753 | 0.15669 | 0.04928 | -0.03356 |
| 31 | Isoamyl butyrate | 0.13699 | 0.00438 | 0.00925 | 0.01689 |
| 32 | Ethenylbenzene | -0.09943 | 0.04166 | -0.13161 | -0.03183 |
| 33 | 2-Propenylbenzene | 0.14601 | 0.01321 | 0.00881 | 0.03089 |
| 34 | Hexyl acetate | -0.02248 | 0.12701 | 0.16728 | -0.15789 |
| 35 | Isoamyl-2-methyl butyrate | 0.04262 | -0.00110 | 0.00217 | 0.00404 |
| 36 | 5-Methyl-2-hexanol | -0.03955 | -0.13343 | 0.10418 | 0.06764 |
| 37 | 2-Octanone | -0.08067 | 0.20468 | -0.05133 | 0.00359 |
| 38 | Isoamyl isovalerate | 0.04614 | -0.00115 | 0.00177 | 0.00474 |
| 39 | Trans-2-(2-Pentenyl)furan | -0.06574 | -0.14944 | 0.11928 | 0.13089 |
| 40 | Acetoin | -0.04748 | 0.01104 | -0.14998 | -0.06291 |
| 41 | Hexanenitrile | -0.03559 | 0.14378 | -0.04779 | 0.18109 |
| 42 | 2-Heptanol | -0.05580 | -0.19862 | 0.08185 | 0.03516 |
| 43 | Prenol | 0.17898 | 0.01320 | 0.00505 | 0.03138 |
| 44 | 2-Heptenal | -0.02809 | 0.12148 | -0.05485 | 0.19619 |
| 45 | 4-Pentenyl butyrate | 0.11945 | 0.00599 | -0.00012 | 0.02406 |
| 46 | 6-Methyl-5-hepten-2-one | -0.11687 | -0.00649 | -0.12417 | -0.02349 |
| 47 | 3-methylcyclopentanol | -0.02451 | -0.09942 | 0.07672 | 0.07670 |
| 48 | 1-Hexanol | 0.06232 | 0.17290 | -0.05569 | 0.17059 |
| 49 | 2-Isopropyl pyrazine | -0.01554 | -0.06312 | -0.12506 | -0.15754 |
| 50 | 6-Methyl-2-heptanol | -0.03825 | -0.11719 | -0.12369 | -0.15237 |
| 51 | Heptyl acetate | -0.02629 | 0.14259 | 0.16049 | -0.15112 |
| 52 | Methyl octanoate | -0.01503 | -0.04362 | 0.01466 | 0.00829 |
| 53 | 3-Hexen-1-ol, (Z)- | 0.09059 | -0.00975 | 0.01464 | 0.03164 |
| 54 | 3-Octanol | -0.04550 | 0.04189 | 0.06436 | 0.00573 |
| 55 | 2-Nonanone | -0.07506 | 0.03267 | -0.13392 | -0.14149 |
| 56 | Nonanal | -0.02130 | 0.04260 | -0.12310 | 0.08430 |
| 57 | Dimethyl trisulfide | -0.04213 | 0.13465 | 0.10417 | -0.07065 |
| 58 | 2-Methyl-3-isopropylpyrazine | -0.01458 | -0.04512 | -0.09373 | -0.11279 |
| 59 | 1-Nitropentane | -0.04353 | 0.13348 | -0.13220 | 0.18062 |
| 60 | 2-Hexen-1-ol | 0.00548 | 0.10291 | -0.11390 | 0.18415 |
| 61 | Hexyl butanoate | 0.09704 | 0.00598 | 0.00757 | 0.02389 |
| 62 | Perillene | 0.18326 | 0.01002 | 0.00729 | 0.02641 |
| 63 | Benzene,m-di-tert-butyl | -0.02304 | 0.05942 | 0.03462 | 0.00454 |
| 64 | Ethyl octanoate | 0.00327 | -0.03655 | -0.00110 | -0.00207 |
| 65 | 2-Methoxy-3-(methylethyl)-pyrazine | -0.04825 | -0.05162 | -0.23362 | -0.16800 |
| 66 | 2-Octenal | -0.02815 | 0.12328 | -0.05497 | 0.19847 |
| 67 | 1-Octen-3-ol | -0.05352 | 0.14956 | -0.03989 | 0.13145 |
| 68 | Linalool oxide | 0.18406 | 0.01036 | 0.00555 | 0.02754 |
| 69 | 1-Heptanol | 0.03254 | 0.10176 | 0.03312 | -0.01254 |
| 70 | 6-Methyl-5-hepten-2-ol | 0.03865 | -0.00789 | -0.04647 | -0.15121 |
| 71 | Octyl acetate | -0.02761 | 0.14592 | 0.15340 | -0.14415 |
| 72 | 2-Ethyl-1-hexanol | -0.01334 | 0.12663 | -0.02513 | -0.08972 |
| 73 | Copaene | 0.16358 | 0.01045 | -0.01367 | 0.03029 |
| 74 | 2,5-Bis(1-methylethyl)pyrazine | -0.01508 | -0.05989 | -0.12359 | -0.16158 |
| 75 | Methyl 2-hydroxy-3-methyl pentanoate | -0.03897 | -0.07883 | -0.04018 | -0.01003 |
| 76 | 1-Nitrohexane | -0.04671 | 0.18622 | -0.02434 | 0.16209 |
| 77 | 2,4-Heptadienal | -0.02020 | 0.04153 | -0.11836 | 0.08383 |
| 78 | 2-Nonanol | 0.17715 | 0.00396 | 0.00107 | -0.00092 |
| 79 | 3,3-Dimethylcyclohexanol | -0.04244 | -0.14112 | 0.10888 | 0.07385 |
| 80 | 2-Isobutyl-3-methoxypyrazine | -0.05023 | -0.04977 | -0.24172 | -0.16023 |
| 81 | Linalool | 0.18152 | 0.00874 | 0.00495 | 0.02381 |
| 82 | Benzaldehyde | -0.03098 | 0.08647 | -0.13106 | 0.15421 |
| 83 | Dihydro-2-methyl-3(2H)-thiophenone | -0.04566 | 0.12088 | 0.07126 | -0.04535 |
| 84 | 1-Octanol | 0.10762 | 0.08252 | -0.10969 | -0.00666 |
| 85 | Nonyl acetate | 0.00848 | 0.08621 | 0.14538 | -0.12639 |
| 86 | Acetic acid | 0.06338 | 0.00296 | 0.02550 | -0.00725 |
| 87 | Methyl decanoate | -0.01733 | -0.04609 | 0.02933 | 0.02408 |
| 88 | 2-Undecanone | -0.06085 | 0.08315 | 0.04910 | -0.05275 |
| 89 | 6-Methyl-3,5-heptadien-2-one | -0.02554 | -0.06773 | 0.04521 | 0.04220 |
| 90 | 4-Terpineol | -0.02745 | -0.02025 | -0.10445 | -0.06124 |
| 91 | Benzonitrile | -0.05639 | 0.10179 | -0.17976 | 0.11154 |
| 92 | Methyl benzoate | 0.15840 | 0.01086 | 0.03206 | 0.01150 |
| 93 | 3-(Methylthio)propyl acetate | -0.01577 | 0.09729 | 0.15487 | -0.14600 |
| 94 | 1-Nonanol | 0.15772 | -0.03044 | -0.04577 | 0.01702 |
| 95 | Isopropyl benzoate | 0.08198 | 0.00846 | 0.04096 | -0.00293 |
| 96 | Acetophenone | -0.12536 | 0.11914 | -0.00453 | 0.01922 |
| 97 | Ethyl benzoate | 0.10307 | 0.01036 | 0.03757 | -0.00274 |
| 98 | Disulfide, methyl (methylthio)methyl | -0.01587 | 0.01147 | 0.02931 | 0.00168 |
| 99 | Butanoic acid | 0.13376 | 0.00483 | 0.02922 | 0.00342 |
| 100 | Ionene | -0.02448 | -0.04751 | 0.04345 | 0.03538 |
| 101 | Pentanoic acid | -0.02427 | -0.07993 | 0.06208 | 0.03665 |
| 102 | 3-(Methylthio)-1-propanol | -0.05095 | 0.17146 | 0.08960 | -0.06380 |
| 103 | Benzyl acetate | 0.10226 | 0.03572 | 0.06872 | -0.03365 |
| 104 | sec-Butyl benzoate | 0.08313 | 0.00750 | 0.04408 | -0.00183 |
| 105 | Isopropyl phenylacetate | 0.04446 | 0.01405 | -0.01174 | 0.03891 |
| 106 | Propyl benzoate | 0.08872 | 0.00860 | 0.04581 | -0.00414 |
| 107 | Methyl phenylacetate | 0.04835 | 0.01497 | -0.01295 | 0.04075 |
| 108 | Ethyl phenylacetate | 0.04151 | 0.01366 | -0.01076 | 0.03757 |
| 109 | Methyl salicylate | 0.18290 | 0.00968 | 0.00052 | 0.02374 |
| 110 | 2-Tridecanone | -0.03279 | 0.09335 | 0.01067 | -0.03505 |
| 111 | Phenethyl acetate | -0.01403 | 0.10093 | 0.15326 | -0.15474 |
| 112 | Ethyl salicylate | 0.14045 | 0.00456 | -0.01826 | 0.01169 |
| 113 | 1-Phenyl-2-propanol | -0.06158 | -0.19727 | 0.14907 | 0.11755 |
| 114 | Geranylacetone | -0.08292 | 0.16063 | 0.09611 | 0.06229 |
| 115 | Guaiacol | 0.04006 | -0.05009 | -0.13918 | -0.17416 |
| 116 | Benzyl alcohol | 0.18405 | 0.02369 | -0.00322 | 0.03840 |
| 117 | Pentyl benzoate | 0.08500 | 0.00855 | 0.04602 | -0.00414 |
| 118 | Phenylethyl alcohol | 0.13996 | -0.00633 | -0.06515 | -0.04937 |
| 119 | Phenethyl butyrate | 0.09537 | 0.00517 | -0.00212 | 0.01861 |
| 120 | Creosol | -0.07600 | -0.19541 | 0.12324 | 0.06801 |
| 121 | 3-Methyl-3-butenyl benzoate | 0.06158 | 0.00783 | 0.04177 | -0.01117 |
| 122 | Isoamyl phenylacetate | 0.03674 | 0.01268 | -0.00929 | 0.03484 |
| 123 | Phenol | 0.13776 | -0.04075 | -0.10269 | -0.10883 |
| 124 | 4-Ethylguaiacol | 0.11250 | -0.02733 | -0.06848 | -0.09586 |
| 125 | Benzenepropanol | 0.18090 | 0.01133 | 0.00813 | 0.02602 |
| 126 | 4-Methylphenol | 0.06166 | 0.01060 | 0.02159 | -0.01404 |
| 127 | Eugenol | -0.06360 | -0.21330 | 0.07628 | 0.03260 |
| 128 | 4-Ethylphenol | 0.17648 | 0.00766 | 0.01364 | 0.01922 |
| 129 | 2-Methoxy-4-vinylphenol | 0.09124 | 0.05013 | -0.00778 | -0.01982 |
| 130 | Ethyl palmitate | 0.18099 | 0.01013 | 0.01654 | 0.02197 |

^1^The compounds present only once across all cactus-microorganism combinations were excluded from the PCA. ^2^Compounds co-eluting at the same time on the column.

**Table S4.** Principal component values for volatile compounds from all four host cacti inoculated with the yeast *Candida sonorensis*^1^.

|  |  | **PC 1** | **PC 2** | **PC3** | **PC 4** |
| --- | --- | --- | --- | --- | --- |
|  | **Eigenvalue** | 43.94 | 18.78 | 16.10 | 2.11 |
|  | **Percent variation** | 51.10 | 21.83 | 18.71 | 2.45 |
|  | **Eigenvectors** |  |  |  |  |
| 3 | Isopropyl alcohol | 0.14904 | 0.02801 | 0.01152 | -0.00795 |
| 4 | 2-Butanol | 0.14848 | 0.02800 | 0.1229 | 0.04318 |
| 5 | Ethyl butanoate | -0.03860 | -0.16389 | 0.14044 | -0.00272 |
| 6 | Ethyl 2-methylbutanoate | 0.14917 | 0.02810 | 0.01173 | 0.01349 |
| 7 | Methyl thioacetate | -0.03175 | -0.03316 | -0.17565 | -0.41293 |
| 8 | Ethyl isovalerate | 0.13188 | 0.02513 | 0.01191 | 0.12941 |
| 9 | Dimethyl disulfide | -0.03980 | -0.04812 | -0.23207 | -0.06082 |
| 13 | Isoamyl acetate/Pentyl isobutyrate^2^ | 0.10682 | -0.00341 | -0.12614 | -0.15445 |
| 17 | 4-Methyl-2-pentanol | -0.06841 | 0.19252 | 0.07220 | 0.03497 |
| 19 | 2-Heptanone | -0.06079 | -0.16071 | 0.14470 | 0.02232 |
| 20 | Isoamyl propionate | 0.13244 | 0.02456 | 0.00975 | -0.09024 |
| 21 | 3-Hexanol | -0.06856 | 0.19344 | 0.07272 | 0.03758 |
| 22 | Isoamyl alcohol/2-methyl-1-butanol^2^ | 0.06252 | -0.10695 | -0.18415 | 0.14380 |
| 24 | 2-Pentylfuran | -0.06649 | -0.05044 | -0.20314 | 0.15843 |
| 26 | 6-Methyl-2-heptanone | -0.05651 | -0.13721 | 0.16721 | 0.01761 |
| 29 | Isobutenylcarbinol | 0.14478 | -0.01721 | 0.06377 | 0.04098 |
| 30 | 3-Octanone | -0.08441 | 0.08299 | -0.17727 | 0.01257 |
| 31 | Isoamyl butyrate | 0.14456 | 0.02699 | 0.01119 | -0.03989 |
| 32 | Ethenylbenzene | -0.04085 | -0.17327 | 0.14877 | 0.00799 |
| 33 | 2-Propenylbenzene | 0.13254 | 0.02516 | 0.01254 | 0.13554 |
| 36 | 5-Methyl-2-hexanol | -0.06854 | 0.19333 | 0.07270 | 0.03832 |
| 37 | 2-Octanone | -0.06844 | -0.18751 | -0.08031 | 0.08304 |
| 39 | Trans-2-(2-Pentenyl)furan | -0.06849 | 0.19336 | 0.07273 | 0.03817 |
| 40 | Acetoin | -0.04692 | -0.18043 | 0.10895 | 0.13188 |
| 42 | 2-Heptanol | -0.06857 | 0.19344 | 0.07271 | 0.03728 |
| 43 | Prenol | 0.14932 | 0.02805 | 0.01177 | 0.00013 |
| 45 | 4-Pentenyl butyrate | 0.14258 | 0.02687 | 0.01289 | 0.08448 |
| 46 | 6-Methyl-5-hepten-2-one | -0.09789 | -0.07786 | 0.16438 | 0.05138 |
| 48 | 1-Hexanol | 0.14692 | -0.00955 | -0.02941 | 0.03769 |
| 50 | 6-Methyl-2-heptanol | -0.09148 | -0.00072 | 0.19130 | 0.04949 |
| 52 | Methyl octanoate | -0.06722 | 0.19029 | 0.07185 | 0.04271 |
| 53 | 3-Hexen-1-ol, (Z)- | 0.14799 | 0.02785 | 0.01110 | -0.01433 |
| 54 | 3-Octanol | -0.06781 | 0.19200 | 0.07234 | 0.03863 |
| 55 | 2-Nonanone | -0.05270 | -0.12232 | 0.05653 | 0.10679 |
| 57 | Dimethyl trisulfide | -0.02909 | -0.04156 | -0.18127 | 0.40129 |
| 59 | 1-Nitropentane | -0.03619 | -0.15771 | 0.13591 | 0.01174 |
| 62 | Perillene | 0.14742 | 0.02763 | 0.01224 | 0.01314 |
| 63 | Benzene,m-di-tert-butyl | -0.03937 | -0.04765 | -0.22924 | -0.05911 |
| 64 | Ethyl octanoate | 0.14409 | 0.02706 | 0.01014 | -0.05071 |
| 65 | 2-Methoxy-3-(methylethyl)-pyrazine | -0.03989 | -0.16876 | 0.14520 | 0.01982 |
| 67 | 1-Octen-3-ol | -0.03711 | -0.04231 | -0.21284 | -0.22937 |
| 68 | Linalool oxide | 0.14934 | 0.02808 | 0.01211 | 0.01808 |
| 69 | 1-Heptanol | 0.14776 | 0.02779 | 0.01255 | 0.04232 |
| 70 | 6-Methyl-5-hepten-2-ol | 0.11704 | 0.05134 | 0.08070 | -0.18582 |
| 72 | 2-Ethyl-1-hexanol | 0.09731 | -0.11799 | 0.13268 | 0.00074 |
| 73 | Copaene | 0.14328 | 0.00928 | 0.02909 | 0.07104 |
| 75 | Methyl 2-hydroxy-3-methyl pentanoate | -0.09066 | -0.01586 | 0.19550 | 0.03628 |
| 76 | 1-Nitrohexane | -0.05152 | -0.10247 | -0.19238 | 0.12894 |
| 78 | 2-Nonanol | 0.12504 | 0.01558 | -0.03692 | -0.17801 |
| 79 | 3,3-Dimethylcyclohexanol | -0.06844 | 0.19308 | 0.07265 | 0.03933 |
| 80 | 2-Isobutyl-3-methoxypyrazine | -0.04085 | -0.17356 | 0.14910 | 0.01019 |
| 81 | Linalool | 0.14869 | 0.02789 | 0.01170 | -0.00975 |
| 82 | Benzaldehyde | -0.03624 | -0.15831 | 0.13625 | 0.00467 |
| 83 | Dihydro-2-methyl-3(2H)-thiophenone | -0.04445 | -0.03716 | -0.23185 | 0.03593 |
| 84 | 1-Octanol | 0.14204 | -0.05673 | -0.01692 | 0.05290 |
| 87 | Methyl decanoate | -0.06120 | 0.17341 | 0.06481 | 0.01989 |
| 88 | 2-Undecanone | -0.11169 | 0.10996 | -0.10008 | 0.11569 |
| 89 | 6-Methyl-3,5-heptadien-2-one | -0.06744 | 0.19064 | 0.07195 | 0.04291 |
| 90 | 4-Terpineol | -0.04107 | -0.17350 | 0.14897 | 0.01051 |
| 91 | Benzonitrile | -0.04520 | -0.17309 | 0.09188 | 0.04247 |
| 92 | Methyl benzoate | 0.14891 | 0.02802 | 0.01233 | 0.03211 |
| 94 | 1-Nonanol | 0.14555 | -0.04016 | 0.04061 | 0.01862 |
| 95 | Isopropyl benzoate | 0.14603 | 0.02763 | 0.01196 | 0.05629 |
| 96 | Acetophenone | -0.12757 | -0.05857 | -0.09629 | -0.04627 |
| 97 | Ethyl benzoate | 0.14885 | 0.02806 | 0.01219 | 0.03661 |
| 99 | Butanoic acid | 0.13895 | 0.02599 | 0.00937 | -0.08657 |
| 101 | Pentanoic acid | -0.06656 | 0.18824 | 0.07114 | 0.04471 |
| 102 | 3-(Methylthio)-1-propanol | -0.03533 | -0.04702 | -0.21458 | 0.24941 |
| 106 | Propyl benzoate | 0.14762 | 0.02779 | 0.01101 | -0.01526 |
| 109 | Methyl salicylate | 0.14956 | 0.02559 | 0.01466 | 0.01892 |
| 110 | 2-Tridecanone | -0.03955 | -0.04873 | -0.23207 | 0.00195 |
| 112 | Ethyl salicylate | 0.14894 | 0.02802 | 0.01232 | 0.03057 |
| 113 | 1-Phenyl-2-propanol | -0.06844 | 0.19272 | 0.07228 | 0.03452 |
| 114 | Geranylacetone | -0.09250 | 0.13529 | -0.11699 | 0.13272 |
| 115 | Guaiacol | 0.11663 | 0.00912 | -0.07461 | 0.38239 |
| 116 | Benzyl alcohol | 0.14962 | 0.02668 | 0.00367 | 0.02192 |
| 118 | Phenylethyl alcohol | 0.12745 | -0.08803 | 0.05090 | 0.18554 |
| 120 | Creosol | -0.08357 | 0.19029 | 0.01067 | 0.04823 |
| 123 | Phenol | 0.14727 | 0.01601 | -0.00566 | 0.12634 |
| 124 | 4-Ethylguaiacol | 0.13565 | 0.02474 | 0.00409 | -0.00152 |
| 125 | Benzenepropanol | 0.14934 | 0.02811 | 0.01172 | 0.00732 |
| 126 | 4-Methylphenol | 0.14868 | 0.02801 | 0.01234 | 0.03997 |
| 127 | Eugenol | -0.06802 | 0.19228 | 0.07248 | 0.04073 |
| 128 | 4-Ethylphenol | 0.12621 | 0.02332 | 0.00975 | -0.08343 |
| 129 | 2-Methoxy-4-vinylphenol | 0.13342 | 0.02445 | -0.03558 | -0.03968 |
| 130 | Ethyl palmitate | 0.14854 | 0.02792 | 0.01128 | -0.01457 |

^1^The compounds present only once across all cactus-microorganism combinations were excluded from the PCA. ^2^Compounds co-eluting at the same time on the column.

**Table S5.** Principal component values for volatile compounds from all four host cacti inoculated with the yeast *Candida valida*^1^.

|  |  | **PC 1** | **PC 2** | **PC3** | **PC 4** |
| --- | --- | --- | --- | --- | --- |
|  | **Eigenvalue** | 38.15 | 13.36 | 9.41 | 5.02 |
|  | **Percent variation** | 51.55 | 18.06 | 12.71 | 6.79 |
|  | **Eigenvectors** |  |  |  |  |
| 1 | Octane | -0.07873 | 0.00453 | 0.21081 | 0.00376 |
| 3 | Isopropyl alcohol | 0.15912 | 0.00743 | 0.01565 | -0.07089 |
| 4 | 2-Butanol | 0.13280 | 0.00414 | 0.01580 | 0.24824 |
| 8 | Ethyl isovalerate | 0.16060 | 0.00596 | 0.01595 | -0.01727 |
| 9 | Dimethyl disulfide | -0.04637 | -0.05394 | -0.29615 | 0.01207 |
| 13 | Isoamyl acetate/Pentyl isobutyrate^2^ | 0.15382 | 0.00415 | 0.01567 | 0.06120 |
| 17 | 4-Methyl-2-pentanol | -0.04507 | -0.18801 | 0.19326 | 0.00119 |
| 19 | 2-Heptanone | -0.06303 | 0.23235 | 0.10008 | 0.00632 |
| 21 | 3-Hexanol | -0.03873 | -0.15417 | 0.17282 | -0.00069 |
| 22 | Isoamyl alcohol/2-methyl-1-butanol^2^ | 0.15128 | 0.00876 | 0.03566 | -0.01919 |
| 24 | 2-Pentylfuran | -0.08565 | -0.10314 | -0.23070 | 0.01439 |
| 25 | Ethyl caproate | 0.12331 | 0.00692 | 0.01587 | 0.26143 |
| 26 | 6-Methyl-2-heptanone | -0.06657 | 0.22238 | 0.12570 | 0.00605 |
| 29 | Isobutenylcarbinol | 0.15517 | 0.01220 | 0.03175 | -0.03749 |
| 30 | 3-Octanone | -0.05957 | 0.23457 | 0.10463 | 0.00578 |
| 31 | Isoamyl butyrate | 0.10316 | 0.00070 | 0.00670 | -0.25745 |
| 32 | Ethenylbenzene | -0.03473 | 0.21601 | 0.12698 | -0.01583 |
| 33 | 2-Propenylbenzene | 0.15880 | 0.00761 | 0.01567 | -0.07063 |
| 36 | 5-Methyl-2-hexanol | -0.04116 | -0.17648 | 0.17211 | 0.00219 |
| 37 | 2-Octanone | -0.07798 | 0.22422 | 0.00553 | 0.01071 |
| 39 | Trans-2-(2-Pentenyl)furan | -0.05398 | -0.19046 | 0.17770 | 0.00111 |
| 40 | Acetoin | -0.04861 | 0.19786 | 0.08842 | 0.00497 |
| 42 | 2-Heptanol | -0.04513 | -0.18741 | 0.19430 | 0.00099 |
| 43 | Prenol | 0.15681 | 0.00850 | 0.01604 | -0.04295 |
| 45 | 4-Pentenyl butyrate | 0.09154 | 0.00058 | 0.00515 | -0.30094 |
| 46 | 6-Methyl-5-hepten-2-one | -0.10324 | 0.07256 | 0.22499 | 0.00669 |
| 48 | 1-Hexanol | 0.15178 | 0.00879 | 0.01513 | -0.09341 |
| 53 | 3-Hexen-1-ol, (Z)- | 0.15649 | 0.00797 | 0.01536 | -0.08995 |
| 55 | 2-Nonanone | -0.06468 | 0.20328 | 0.12055 | 0.00566 |
| 57 | Dimethyl trisulfide | -0.03585 | -0.03813 | -0.21935 | 0.01106 |
| 62 | Perillene | 0.16149 | 0.00680 | 0.01661 | 0.01526 |
| 64 | Ethyl octanoate | 0.16155 | 0.00691 | 0.01657 | 0.00748 |
| 65 | 2-Methoxy-3-(methylethyl)-pyrazine | -0.05971 | 0.23298 | 0.10310 | 0.00572 |
| 68 | Linalool oxide | 0.16109 | 0.00743 | 0.01659 | -0.00024 |
| 69 | 1-Heptanol | 0.15799 | 0.00746 | 0.01538 | -0.08713 |
| 70 | 6-Methyl-5-hepten-2-ol | 0.13677 | -0.07963 | -0.08775 | -0.05643 |
| 72 | 2-Ethyl-1-hexanol | 0.10358 | 0.17812 | 0.09135 | -0.04144 |
| 73 | Copaene | 0.15602 | 0.00754 | 0.01499 | -0.10866 |
| 76 | 1-Nitrohexane | -0.04343 | -0.05186 | -0.28127 | 0.01057 |
| 78 | 2-Nonanol | 0.15136 | 0.00550 | 0.01682 | 0.15257 |
| 80 | 2-Isobutyl-3-methoxypyrazine | -0.05916 | 0.22684 | 0.10133 | 0.00557 |
| 81 | Linalool | 0.15948 | 0.00683 | 0.01550 | -0.07196 |
| 83 | Dihydro-2-methyl-3(2H)-thiophenone | -0.06539 | -0.16586 | -0.11216 | 0.01213 |
| 84 | 1-Octanol | 0.15849 | 0.00818 | 0.01616 | -0.03773 |
| 88 | 2-Undecanone | -0.05851 | 0.23268 | 0.10259 | 0.00572 |
| 89 | 6-Methyl-3,5-heptadien-2-one | -0.04226 | -0.10747 | 0.16644 | -0.00069 |
| 90 | 4-Terpineol | -0.04238 | 0.15976 | 0.08174 | 0.00409 |
| 91 | Benzonitrile | -0.08444 | 0.18832 | -0.09176 | 0.01301 |
| 92 | Methyl benzoate | 0.13673 | 0.00410 | 0.01592 | 0.22788 |
| 94 | 1-Nonanol | 0.16134 | 0.00649 | 0.01617 | -0.01625 |
| 95 | Isopropyl benzoate | 0.09464 | 0.00096 | 0.01243 | 0.33692 |
| 96 | Acetophenone | -0.13281 | 0.00033 | -0.15478 | 0.01909 |
| 97 | Ethyl benzoate | 0.10598 | 0.00174 | 0.01344 | 0.31566 |
| 98 | Disulfide, methyl (methylthio)methyl | -0.04632 | -0.05391 | -0.29598 | 0.01201 |
| 99 | Butanoic acid | 0.11102 | 0.00104 | 0.01325 | 0.27499 |
| 100 | Ionene | -0.05273 | -0.19066 | 0.17586 | 0.00156 |
| 106 | Propyl benzoate | 0.09550 | 0.00102 | 0.01251 | 0.33550 |
| 109 | Methyl salicylate | 0.15877 | 0.00914 | 0.01719 | -0.07278 |
| 112 | Ethyl salicylate | 0.16063 | 0.00702 | 0.01692 | 0.04463 |
| 113 | 1-Phenyl-2-propanol | -0.04448 | -0.18712 | 0.18935 | 0.00153 |
| 114 | Geranylacetone | -0.05400 | -0.18672 | 0.17509 | 0.00078 |
| 115 | Guaiacol | 0.15900 | 0.00590 | 0.01653 | -0.07921 |
| 116 | Benzyl alcohol | 0.16112 | 0.00488 | 0.01860 | -0.02839 |
| 118 | Phenylethyl alcohol | 0.15332 | 0.02370 | -0.00197 | 0.01668 |
| 119 | Phenethyl butyrate | 0.08715 | 0.00054 | 0.00459 | -0.31497 |
| 120 | Creosol | -0.04435 | -0.18677 | 0.18856 | 0.00158 |
| 123 | Phenol | 0.16099 | 0.01010 | 0.01477 | -0.03182 |
| 124 | 4-Ethylguaiacol | 0.14146 | 0.00321 | 0.01215 | -0.13802 |
| 125 | Benzenepropanol | 0.15919 | 0.00790 | 0.01691 | 0.03337 |
| 126 | 4-Methylphenol | 0.15916 | 0.00689 | 0.01543 | -0.07718 |
| 127 | Eugenol | -0.04495 | -0.18801 | 0.19232 | 0.00130 |
| 128 | 4-Ethylphenol | 0.15027 | 0.00368 | 0.01390 | -0.06101 |
| 129 | 2-Methoxy-4-vinylphenol | 0.13951 | -0.00143 | 0.02583 | 0.15165 |
| 130 | Ethyl palmitate | 0.13407 | 0.00109 | 0.01269 | 0.02914 |

^1^The compounds present only once across all cactus-microorganism combinations were excluded from the PCA. ^2^Compounds co-eluting at the same time on the column.

**Table S6.** Principal component values for volatile compounds in all four host cacti inoculated with the yeast *Dipodascus starmeri*^1^.

|  |  | **PC 1** | **PC 2** | **PC3** | **PC 4** |
| --- | --- | --- | --- | --- | --- |
|  | **Eigenvalue** | 37.07 | 16.65 | 14.73 | 3.68 |
|  | **Percent variation** | 46.34 | 20.82 | 18.42 | 4.60 |
|  | **Eigenvectors** |  |  |  |  |
| 2 | Acetone | -0.06799 | 0.12520 | 0.11350 | -0.28561 |
| 3 | Isopropyl alcohol | 0.15622 | 0.00647 | 0.00481 | -0.01666 |
| 4 | 2-Butanol | 0.16303 | 0.00621 | 0.00372 | -0.00382 |
| 6 | Ethyl 2-methylbutanoate | 0.12895 | 0.00323 | -0.00046 | 0.03228 |
| 8 | Ethyl isovalerate | 0.15784 | 0.00638 | 0.00459 | -0.01410 |
| 9 | Dimethyl disulfide | -0.03426 | -0.10129 | 0.13336 | 0.30683 |
| 13 | Isoamyl acetate/Pentyl isobutyrate^2^ | 0.14988 | -0.03333 | 0.05428 | 0.11877 |
| 15 | Butanoic acid, 1-methylpropyl ester | 0.14331 | 0.00648 | 0.00567 | -0.02843 |
| 17 | 4-Methyl-2-pentanol | -0.05301 | -0.08342 | -0.22634 | -0.01109 |
| 19 | 2-Heptanone | -0.06652 | 0.20750 | 0.06590 | 0.03932 |
| 22 | Isoamyl alcohol/2-methyl-1-butanol^2^ | 0.15693 | -0.00932 | 0.06725 | 0.04258 |
| 24 | 2-Pentylfuran | -0.09223 | -0.17807 | 0.06948 | 0.10028 |
| 25 | Ethyl caproate | -0.03829 | 0.15974 | 0.03140 | 0.00949 |
| 26 | 6-Methyl-2-heptanone | -0.05721 | 0.21993 | 0.04179 | 0.02626 |
| 29 | Isobutenylcarbinol | 0.13995 | 0.11341 | 0.03525 | 0.04246 |
| 30 | 3-Octanone | -0.06401 | -0.07811 | 0.19837 | -0.07120 |
| 31 | Isoamyl butyrate | 0.15349 | 0.00477 | 0.00129 | 0.01941 |
| 32 | Ethenylbenzene | -0.11562 | -0.05384 | 0.14602 | 0.12311 |
| 33 | 2-Propenylbenzene | 0.15980 | 0.00636 | 0.00409 | -0.00837 |
| 36 | 5-Methyl-2-hexanol | -0.05306 | -0.08342 | -0.22638 | -0.01087 |
| 37 | 2-Octanone | -0.06379 | -0.08080 | 0.19810 | -0.09394 |
| 39 | Trans-2-(2-Pentenyl)furan | -0.05292 | -0.08344 | -0.22588 | -0.01067 |
| 40 | Acetoin | -0.06388 | 0.21418 | 0.05716 | 0.05612 |
| 41 | Hexanenitrile | -0.04272 | -0.10994 | 0.15749 | -0.30324 |
| 42 | 2-Heptanol | -0.08587 | 0.03412 | -0.21289 | 0.00635 |
| 43 | Prenol | 0.16372 | 0.00593 | 0.00295 | 0.00432 |
| 45 | 4-Pentenyl butyrate | 0.15313 | 0.00475 | 0.00129 | 0.01926 |
| 46 | 6-Methyl-5-hepten-2-one | -0.09216 | 0.13306 | 0.14620 | -0.00671 |
| 48 | 1-Hexanol | 0.10259 | -0.10961 | 0.13732 | 0.11623 |
| 49 | 2-Isopropyl pyrazine | -0.05107 | 0.18418 | 0.03595 | 0.02784 |
| 50 | 6-Methyl-2-heptanol | -0.07797 | 0.21069 | -0.03024 | 0.03033 |
| 54 | 3-Octanol | -0.05464 | -0.14235 | 0.07075 | 0.04449 |
| 55 | 2-Nonanone | -0.08024 | 0.17274 | 0.11931 | 0.01948 |
| 57 | Dimethyl trisulfide | -0.03843 | -0.11696 | 0.13907 | 0.33928 |
| 58 | 2-Methyl-3-isopropylpyrazine | -0.05859 | 0.22137 | 0.04039 | 0.03168 |
| 59 | 1-Nitropentane | -0.04779 | -0.13833 | 0.17485 | 0.19070 |
| 62 | Perillene | 0.16123 | 0.00623 | 0.00366 | -0.00355 |
| 64 | Ethyl octanoate | 0.16377 | 0.00593 | 0.00309 | 0.00286 |
| 65 | 2-Methoxy-3-(methylethyl)-pyrazine | -0.05871 | 0.22480 | 0.04064 | 0.03094 |
| 67 | 1-Octen-3-ol | -0.03741 | -0.09740 | 0.12795 | -0.34456 |
| 68 | Linalool oxide | 0.16380 | 0.00587 | 0.00291 | 0.00474 |
| 69 | 1-Heptanol | 0.16390 | 0.00601 | 0.00320 | 0.00180 |
| 70 | 6-Methyl-5-hepten-2-ol | -0.02214 | 0.04950 | 0.18092 | 0.08408 |
| 72 | 2-Ethyl-1-hexanol | -0.05238 | -0.14797 | 0.18618 | 0.01451 |
| 73 | Copaene | 0.15423 | 0.00517 | 0.00236 | 0.00832 |
| 74 | 2,5-Bis(1-methylethyl)pyrazine | -0.06167 | 0.22172 | 0.05019 | -0.00476 |
| 76 | 1-Nitrohexane | -0.04093 | -0.11948 | 0.15493 | 0.26035 |
| 78 | 2-Nonanol | 0.16327 | 0.00572 | 0.00254 | 0.00854 |
| 79 | 3,3-Dimethylcyclohexanol | -0.05263 | -0.08297 | -0.22543 | -0.01213 |
| 80 | 2-Isobutyl-3-methoxypyrazine | -0.05876 | 0.22498 | 0.04039 | 0.03146 |
| 81 | Linalool | 0.16294 | 0.00616 | 0.00348 | -0.00133 |
| 84 | 1-Octanol | 0.10850 | 0.10432 | 0.13559 | 0.07626 |
| 88 | 2-Undecanone | -0.05746 | 0.22334 | 0.04005 | 0.02925 |
| 91 | Benzonitrile | -0.09051 | 0.09879 | 0.16657 | 0.15790 |
| 92 | Methyl benzoate | 0.16295 | 0.00603 | 0.00314 | 0.00224 |
| 94 | 1-Nonanol | 0.15426 | 0.05432 | 0.01271 | -0.00157 |
| 95 | Isopropyl benzoate | 0.10847 | 0.00261 | -0.00182 | 0.04241 |
| 96 | Acetophenone | -0.07248 | -0.06793 | 0.20751 | -0.13564 |
| 97 | Ethyl benzoate | 0.14787 | 0.01224 | 0.00652 | 0.02608 |
| 99 | Butanoic acid | 0.14506 | 0.00651 | 0.00550 | -0.02622 |
| 102 | 3-(Methylthio)-1-propanol | -0.04887 | -0.13896 | 0.16713 | -0.03153 |
| 106 | Propyl benzoate | 0.11798 | 0.00301 | -0.00143 | 0.04038 |
| 109 | Methyl salicylate | 0.16293 | 0.01526 | 0.00536 | 0.00141 |
| 110 | 2-Tridecanone | -0.04529 | -0.12207 | 0.17453 | -0.03619 |
| 112 | Ethyl salicylate | 0.15951 | 0.04301 | 0.00865 | 0.01525 |
| 113 | 1-Phenyl-2-propanol | -0.05303 | -0.08326 | -0.22625 | -0.01103 |
| 114 | Geranylacetone | -0.04992 | -0.13571 | 0.18649 | -0.06841 |
| 115 | Guaiacol | -0.01356 | 0.22987 | 0.07418 | -0.05764 |
| 116 | Benzyl alcohol | 0.16359 | 0.00609 | 0.01536 | -0.00172 |
| 118 | Phenylethyl alcohol | 0.16004 | 0.00619 | 0.04541 | -0.00629 |
| 119 | Phenethyl butyrate | 0.16096 | 0.00538 | 0.00203 | 0.01330 |
| 120 | Creosol | -0.11268 | -0.02769 | -0.18020 | -0.04803 |
| 123 | Phenol | 0.08137 | 0.20253 | 0.05139 | 0.01774 |
| 124 | 4-Ethylguaiacol | 0.05817 | 0.13244 | 0.03240 | -0.02780 |
| 125 | Benzenepropanol | 0.16377 | 0.00585 | 0.00283 | 0.00557 |
| 126 | 4-Methylphenol | 0.07716 | -0.07151 | 0.11394 | -0.36797 |
| 127 | Eugenol | -0.09272 | 0.09987 | -0.17374 | 0.01750 |
| 128 | 4-Ethylphenol | 0.16287 | 0.00619 | 0.00358 | -0.00235 |
| 129 | 2-Methoxy-4-vinylphenol | 0.03978 | 0.05084 | 0.14643 | -0.30895 |
| 130 | Ethyl palmitate | 0.16359 | 0.00612 | 0.00341 | -0.00047 |

^1^The compounds present only once across all cactus-microorganism combinations were excluded from the PCA. ^2^Compounds co-eluting at the same time on the column.

**Table S7.** Principal component values for volatile compounds for all four host cacti inoculated with the yeast *Erwinia cacticida*^1^.

|  |  | **PC 1** | **PC 2** | **PC3** | **PC 4** |
| --- | --- | --- | --- | --- | --- |
|  | **Eigenvalue** | 42.50 | 20.05 | 13.95 | 6.21 |
|  | **Percent variation** | 47.76 | 22.53 | 15.67 | 6.98 |
|  | **Eigenvectors** |  |  |  |  |
| 1 | Octane | -0.04632 | -0.13825 | 0.19101 | -0.00225 |
| 3 | Isopropyl alcohol | 0.15065 | -0.00510 | -0.01533 | -0.03235 |
| 4 | 2-Butanol | 0.12875 | -0.00450 | -0.01352 | 0.01084 |
| 5 | Ethyl butanoate | 0.15205 | -0.00678 | -0.01282 | -0.03334 |
| 8 | Ethyl isovalerate | 0.10493 | -0.00351 | -0.01095 | 0.05091 |
| 9 | Dimethyl disulfide | -0.07800 | 0.01008 | -0.22165 | 0.00742 |
| 10 | Hexanal | -0.06602 | -0.08303 | -0.21791 | 0.01393 |
| 12 | 3-Pentanol | -0.04660 | 0.20656 | 0.05512 | -0.01409 |
| 13 | Isoamyl acetate/Pentyl isobutyrate^2^ | 0.14936 | -0.00302 | -0.01212 | 0.06021 |
| 14 | 2-Pentanol | -0.04673 | 0.20687 | 0.05534 | -0.01428 |
| 16 | Isobutyl butanoate | 0.15117 | -0.00343 | -0.01283 | 0.04214 |
| 17 | 4-Methyl-2-pentanol | -0.04675 | 0.20687 | 0.05540 | -0.01436 |
| 19 | 2-Heptanone | -0.07309 | -0.13772 | 0.06531 | -0.00141 |
| 20 | Isoamyl propionate | 0.13529 | -0.00079 | -0.00818 | 0.17484 |
| 21 | 3-Hexanol | -0.04670 | 0.20616 | 0.05536 | -0.01455 |
| 22 | Isoamyl alcohol/2-methyl-1-butanol^2^ | 0.15171 | -0.00546 | -0.00680 | -0.00469 |
| 24 | 2-Pentylfuran | -0.06467 | -0.04158 | -0.21578 | 0.02795 |
| 25 | Ethyl caproate | 0.10982 | -0.09108 | 0.09976 | -0.16946 |
| 26 | 6-Methyl-2-heptanone | -0.05474 | -0.09177 | 0.19204 | -0.01080 |
| 27 | Prenyl acetate | 0.11683 | -0.00752 | -0.01706 | -0.23367 |
| 28 | 1-Pentanol | -0.05463 | -0.15011 | 0.17312 | -0.00230 |
| 29 | Isobutenylcarbinol | 0.07515 | 0.03977 | 0.01006 | 0.29542 |
| 30 | 3-Octanone | -0.09090 | 0.09120 | -0.18218 | 0.00123 |
| 31 | Isoamyl butyrate | 0.13896 | -0.00120 | -0.00899 | 0.16024 |
| 32 | Ethenylbenzene | -0.12659 | -0.01656 | 0.03935 | -0.00850 |
| 33 | 2-Propenylbenzene | 0.10787 | -0.00729 | -0.01606 | -0.28007 |
| 37 | 2-Octanone | -0.07537 | -0.11227 | -0.17468 | 0.01426 |
| 39 | Trans-2-(2-Pentenyl)furan | -0.07556 | 0.18266 | -0.05375 | -0.00703 |
| 40 | Acetoin | -0.04972 | -0.13972 | 0.18221 | -0.00526 |
| 41 | Hexanenitrile | -0.05833 | -0.06433 | -0.23314 | 0.01439 |
| 42 | 2-Heptanol | -0.04676 | 0.20671 | 0.05532 | -0.01437 |
| 43 | Prenol | 0.14955 | -0.00505 | -0.01523 | -0.02612 |
| 44 | 2-Heptenal | -0.05865 | -0.06328 | -0.23166 | 0.01228 |
| 45 | 4-Pentenyl butyrate | 0.12521 | 0.00017 | -0.00625 | 0.20849 |
| 46 | 6-Methyl-5-hepten-2-one | -0.10294 | -0.05618 | 0.18323 | -0.01230 |
| 47 | 3-methylcyclopentanol | -0.04675 | 0.20655 | 0.05537 | -0.01446 |
| 48 | 1-Hexanol | -0.04956 | -0.15094 | -0.13149 | 0.07981 |
| 53 | 3-Hexen-1-ol, (Z)- | 0.14039 | 0.05442 | 0.00375 | 0.12577 |
| 55 | 2-Nonanone | -0.09463 | -0.05670 | 0.14929 | -0.01080 |
| 56 | Nonanal | -0.04422 | -0.13453 | 0.18497 | -0.00163 |
| 59 | 1-Nitropentane | -0.08125 | -0.16724 | -0.00103 | 0.00782 |
| 60 | 2-Hexen-1-ol | -0.08609 | -0.17070 | -0.02196 | 0.00731 |
| 61 | Hexyl butanoate | 0.14254 | -0.00419 | -0.01333 | -0.04775 |
| 62 | Perillene | 0.15153 | -0.00495 | -0.01503 | -0.04794 |
| 64 | Ethyl octanoate | 0.09340 | -0.12207 | 0.15085 | -0.01677 |
| 65 | 2-Methoxy-3-(methylethyl)-pyrazine | -0.04467 | -0.13536 | 0.18611 | -0.00266 |
| 66 | 2-Octenal | -0.05839 | -0.06379 | -0.23306 | 0.01377 |
| 67 | 1-Octen-3-ol | -0.05823 | -0.06267 | -0.22947 | 0.01187 |
| 68 | Linalool oxide | 0.15276 | -0.00453 | -0.01458 | -0.00338 |
| 69 | 1-Heptanol | -0.04663 | 0.20674 | 0.05561 | -0.01440 |
| 70 | 6-Methyl-5-hepten-2-ol | 0.13706 | 0.07305 | 0.05058 | 0.08820 |
| 72 | 2-Ethyl-1-hexanol | 0.11406 | -0.00056 | -0.00703 | 0.21709 |
| 73 | Copaene | 0.10983 | 0.00124 | -0.00405 | 0.27949 |
| 75 | Methyl 2-hydroxy-3-methyl pentanoate | -0.04661 | 0.20689 | 0.05548 | -0.01423 |
| 76 | 1-Nitrohexane | -0.05842 | -0.07073 | -0.21481 | 0.01577 |
| 77 | 2,4-Heptadienal | -0.04100 | -0.12713 | 0.17408 | -0.00041 |
| 78 | 2-Nonanol | 0.15186 | -0.00386 | -0.01348 | 0.01574 |
| 80 | 2-Isobutyl-3-methoxypyrazine | -0.04711 | -0.13834 | 0.19129 | -0.00590 |
| 81 | Linalool | 0.15179 | -0.00354 | -0.01304 | 0.04018 |
| 82 | Benzaldehyde | -0.06441 | -0.15053 | 0.06663 | 0.00538 |
| 83 | Dihydro-2-methyl-3(2H)-thiophenone | -0.04551 | 0.20333 | 0.05423 | -0.01339 |
| 84 | 1-Octanol | 0.02771 | -0.12253 | 0.14379 | 0.17451 |
| 88 | 2-Undecanone | -0.12752 | 0.05251 | 0.06452 | -0.01322 |
| 89 | 6-Methyl-3,5-heptadien-2-one | -0.04548 | 0.20290 | 0.05508 | -0.01412 |
| 91 | Benzonitrile | -0.08267 | -0.16721 | 0.01818 | 0.00392 |
| 92 | Methyl benzoate | 0.12274 | -0.00641 | -0.01550 | -0.21480 |
| 94 | 1-Nonanol | 0.08414 | 0.09991 | 0.12999 | 0.19045 |
| 95 | Isopropyl benzoate | 0.08520 | -0.00641 | -0.01346 | -0.29659 |
| 96 | Acetophenone | -0.11340 | -0.00323 | -0.04569 | -0.00171 |
| 97 | Ethyl benzoate | 0.13651 | -0.00626 | -0.01606 | -0.16858 |
| 99 | Butanoic acid | 0.12928 | -0.00686 | -0.01659 | -0.21109 |
| 106 | Propyl benzoate | 0.08649 | -0.00646 | -0.01360 | -0.29582 |
| 109 | Methyl salicylate | 0.15308 | -0.00425 | -0.01387 | 0.00248 |
| 111 | Phenethyl acetate | 0.14711 | -0.00599 | -0.01639 | -0.10227 |
| 112 | Ethyl salicylate | 0.15232 | -0.00373 | -0.01339 | 0.04066 |
| 113 | 1-Phenyl-2-propanol | -0.04675 | 0.20687 | 0.05539 | -0.01435 |
| 114 | Geranylacetone | -0.08348 | 0.03286 | -0.21626 | 0.00585 |
| 115 | Guaiacol | 0.15225 | -0.00185 | -0.01358 | 0.01702 |
| 116 | Benzyl alcohol | 0.15148 | -0.02396 | -0.02576 | -0.00541 |
| 118 | Phenylethyl alcohol | 0.15013 | 0.00933 | -0.00086 | -0.03003 |
| 119 | Phenethyl butyrate | 0.10327 | 0.00185 | -0.00265 | 0.26759 |
| 120 | Creosol | -0.04671 | 0.20697 | 0.05550 | -0.01433 |
| 123 | Phenol | 0.15213 | -0.00932 | -0.01586 | -0.00112 |
| 124 | 4-Ethylguaiacol | 0.15308 | -0.00433 | -0.01427 | 0.00209 |
| 125 | Benzenepropanol | 0.15158 | -0.00505 | -0.01528 | -0.03279 |
| 127 | Eugenol | -0.04669 | 0.20642 | 0.05551 | -0.01453 |
| 128 | 4-Ethylphenol | 0.14613 | -0.00580 | -0.01596 | -0.11341 |
| 129 | 2-Methoxy-4-vinylphenol | 0.13318 | 0.05021 | 0.00390 | 0.10269 |
| 130 | Ethyl palmitate | 0.15210 | -0.00507 | -0.01530 | -0.04242 |

^1^The compounds present only once across all cactus-microorganism combinations were excluded from the PCA. ^2^Compounds co-eluting at the same time on the column.

**Table S8.** Principal component values for volatile compounds for all four host cacti inoculated with the yeast *Starmera amethionina*^1^.

|  |  | **PC 1** | **PC 2** | **PC3** | **PC 4** |
| --- | --- | --- | --- | --- | --- |
|  | **Eigenvalue** | 36.00 | 19.65 | 14.60 | 7.11 |
|  | **Percent variation** | 40.00 | 21.84 | 16.22 | 7.90 |
|  | **Eigenvectors** |  |  |  |  |
| 1 | Octane | -0.04827 | -0.07701 | -0.19348 | -0.11464 |
| 2 | Acetone | -0.03163 | -0.05412 | 0.15723 | -0.12221 |
| 3 | Isopropyl alcohol | 0.10879 | -0.08338 | 0.14827 | -0.06178 |
| 4 | 2-Butanol | 0.15637 | 0.04918 | -0.02145 | 0.01903 |
| 5 | Ethyl butanoate | -0.07631 | -0.05059 | 0.01505 | 0.24766 |
| 6 | Ethyl 2-methylbutanoate | -0.00849 | -0.13219 | 0.18085 | -0.07660 |
| 7 | Methyl thioacetate | -0.06673 | 0.15134 | 0.04944 | -0.01653 |
| 8 | Ethyl isovalerate | 0.13745 | 0.04329 | -0.01836 | 0.01731 |
| 9 | Dimethyl disulfide | -0.07272 | 0.17174 | 0.05551 | -0.01100 |
| 11 | Ethyl carbonate | -0.06288 | -0.16577 | -0.01982 | 0.17113 |
| 13 | Isoamyl acetate/Pentyl isobutyrate^2^ | -0.04048 | 0.19161 | 0.06145 | -0.02157 |
| 15 | Butanoic acid, 1-methylpropyl ester | 0.12060 | 0.03870 | -0.01718 | 0.01612 |
| 16 | Isobutyl butanoate | 0.15396 | 0.04704 | -0.01825 | 0.01761 |
| 17 | 4-Methyl-2-pentanol | -0.05192 | -0.08046 | -0.20921 | -0.11476 |
| 18 | Pentyl acetate | -0.06994 | 0.16770 | 0.05785 | -0.01543 |
| 19 | 2-Heptanone | -0.06729 | -0.09101 | -0.08269 | 0.22900 |
| 20 | Isoamyl propionate | 0.15095 | 0.04797 | -0.02259 | 0.01810 |
| 22 | Isoamyl alcohol/2-methyl-1-butanol^2^ | 0.14389 | 0.10764 | 0.02382 | 0.00602 |
| 24 | 2-Pentylfuran | -0.11899 | 0.13589 | -0.04009 | -0.07475 |
| 25 | Ethyl caproate | 0.03932 | -0.12666 | 0.16838 | 0.07762 |
| 26 | 6-Methyl-2-heptanone | -0.05855 | -0.10754 | -0.08704 | 0.21646 |
| 27 | Prenyl acetate | 0.11568 | 0.03416 | -0.01304 | 0.01078 |
| 29 | Isobutenylcarbinol | 0.15812 | -0.02400 | 0.05770 | 0.04000 |
| 30 | 3-Octanone | -0.09212 | 0.15488 | 0.02900 | -0.01891 |
| 31 | Isoamyl butyrate | 0.15894 | 0.05002 | -0.02211 | 0.01920 |
| 32 | Ethenylbenzene | -0.14179 | -0.00684 | -0.00154 | 0.14931 |
| 34 | Hexyl acetate | -0.07958 | 0.18257 | 0.06873 | -0.03546 |
| 35 | Isoamyl-2-methyl butyrate | 0.10753 | 0.03680 | -0.02169 | 0.01495 |
| 37 | 2-Octanone | -0.09471 | 0.14623 | 0.07998 | 0.14360 |
| 38 | Isoamyl isovalerate | 0.11805 | 0.04021 | -0.02301 | 0.01661 |
| 39 | Trans-2-(2-Pentenyl)furan | -0.05188 | -0.07939 | -0.20949 | -0.10932 |
| 40 | Acetoin | -0.05545 | -0.06348 | 0.10890 | 0.28104 |
| 42 | 2-Heptanol | -0.04512 | -0.19153 | 0.06096 | -0.10132 |
| 43 | Prenol | 0.16140 | 0.04950 | -0.02008 | 0.01816 |
| 45 | 4-Pentenyl butyrate | 0.15118 | 0.04875 | -0.02341 | 0.01928 |
| 46 | 6-Methyl-5-hepten-2-one | -0.12246 | -0.02669 | -0.12735 | 0.08031 |
| 48 | 1-Hexanol | 0.02189 | 0.21413 | 0.05983 | -0.03688 |
| 49 | 2-Isopropyl pyrazine | -0.00947 | -0.13679 | 0.18672 | -0.08831 |
| 50 | 6-Methyl-2-heptanol | -0.00899 | -0.13671 | 0.18690 | -0.08202 |
| 51 | Heptyl acetate | -0.07390 | 0.17353 | 0.06344 | -0.02622 |
| 52 | Methyl octanoate | -0.03663 | -0.13641 | 0.02730 | -0.03168 |
| 54 | 3-Octanol | -0.08027 | 0.18006 | 0.07253 | -0.04806 |
| 55 | 2-Nonanone | -0.08141 | 0.02173 | 0.04112 | 0.31760 |
| 57 | Dimethyl trisulfide | -0.08046 | 0.17838 | 0.06820 | -0.04264 |
| 59 | 1-Nitropentane | -0.08960 | 0.13105 | 0.05647 | 0.21177 |
| 61 | Hexyl butanoate | 0.15937 | 0.04972 | -0.02119 | 0.01893 |
| 62 | Perillene | 0.16038 | 0.04902 | -0.01944 | 0.01802 |
| 64 | Ethyl octanoate | 0.03332 | -0.12041 | 0.20088 | -0.03119 |
| 65 | 2-Methoxy-3-(methylethyl)-pyrazine | -0.03153 | -0.13042 | 0.13226 | 0.22781 |
| 68 | Linalool oxide | 0.16095 | 0.04937 | -0.01989 | 0.01823 |
| 69 | 1-Heptanol | -0.05961 | 0.11620 | 0.06249 | -0.07850 |
| 70 | 6-Methyl-5-hepten-2-ol | 0.00155 | -0.08325 | 0.20212 | -0.15313 |
| 71 | Octyl acetate | -0.07474 | 0.17533 | 0.06372 | -0.02592 |
| 72 | 2-Ethyl-1-hexanol | -0.04394 | 0.09103 | 0.21880 | -0.01435 |
| 73 | Copaene | 0.16163 | 0.04979 | -0.02051 | 0.01839 |
| 74 | 2,5-Bis(1-methylethyl)pyrazine | -0.00924 | -0.13828 | 0.18896 | -0.08484 |
| 76 | 1-Nitrohexane | -0.07523 | 0.17738 | 0.06148 | -0.01962 |
| 78 | 2-Nonanol | 0.16290 | 0.00839 | 0.03280 | 0.02995 |
| 80 | 2-Isobutyl-3-methoxypyrazine | -0.02898 | -0.14214 | 0.15521 | 0.17168 |
| 81 | Linalool | 0.16112 | 0.04929 | -0.01975 | 0.01804 |
| 83 | Dihydro-2-methyl-3(2H)-thiophenone | -0.07520 | -0.01058 | 0.15951 | -0.05836 |
| 84 | 1-Octanol | 0.03894 | -0.00166 | 0.23803 | -0.06095 |
| 88 | 2-Undecanone | -0.10829 | 0.14211 | 0.04867 | 0.02828 |
| 89 | 6-Methyl-3,5-heptadien-2-one | -0.05120 | -0.07873 | -0.20652 | -0.10712 |
| 91 | Benzonitrile | -0.03395 | -0.09114 | 0.06853 | 0.31803 |
| 92 | Methyl benzoate | 0.15933 | 0.04987 | -0.02204 | 0.01865 |
| 94 | 1-Nonanol | 0.15539 | 0.00256 | 0.04121 | 0.01089 |
| 96 | Acetophenone | -0.13432 | 0.05275 | 0.01405 | -0.01806 |
| 97 | Ethyl benzoate | 0.16333 | 0.04105 | -0.00786 | 0.01245 |
| 99 | Butanoic acid | 0.11491 | 0.03211 | -0.00894 | 0.00939 |
| 102 | 3-(Methylthio)-1-propanol | -0.08205 | 0.18305 | 0.07082 | -0.04422 |
| 109 | Methyl salicylate | 0.16221 | 0.04509 | -0.01417 | 0.01729 |
| 110 | 2-Tridecanone | -0.08415 | 0.14507 | 0.05402 | 0.12790 |
| 111 | Phenethyl acetate | -0.03910 | 0.02785 | 0.19837 | -0.10406 |
| 112 | Ethyl salicylate | 0.15951 | 0.05022 | -0.02238 | 0.01914 |
| 113 | 1-Phenyl-2-propanol | -0.05256 | -0.08104 | -0.21193 | -0.11331 |
| 114 | Geranylacetone | -0.11377 | 0.14230 | -0.04706 | -0.10845 |
| 115 | Guaiacol | 0.03476 | -0.11581 | 0.19970 | -0.10377 |
| 116 | Benzyl alcohol | 0.16022 | 0.05471 | -0.01025 | 0.03650 |
| 118 | Phenylethyl alcohol | 0.14164 | -0.04642 | 0.08640 | 0.11681 |
| 119 | Phenethyl butyrate | 0.14187 | 0.04481 | -0.02106 | 0.01645 |
| 120 | Creosol | -0.08546 | -0.10046 | -0.13897 | -0.18659 |
| 123 | Phenol | 0.11997 | -0.07574 | 0.14804 | -0.05810 |
| 124 | 4-Ethylguaiacol | 0.10128 | -0.04159 | 0.11371 | -0.06901 |
| 125 | Benzenepropanol | 0.16122 | 0.04942 | -0.02009 | 0.01805 |
| 126 | 4-Methylphenol | 0.02580 | -0.03719 | 0.15733 | -0.11944 |
| 127 | Eugenol | -0.05101 | -0.17812 | -0.02464 | -0.16835 |
| 128 | 4-Ethylphenol | 0.16102 | 0.04923 | -0.01967 | 0.01802 |
| 129 | 2-Methoxy-4-vinylphenol | -0.00367 | 0.04908 | 0.11621 | -0.10235 |
| 130 | Ethyl palmitate | 0.15791 | 0.04773 | -0.01850 | 0.01692 |

^1^The compounds present only once across all cactus-microorganism combinations were excluded from the PCA. ^2^Compounds co-eluting at the same time on the column.

**Table S9.** Principal component values for volatile compounds for all four host cacti inoculated with the yeast *Sporopachydermia cereana*^1^.

|  |  | **PC 1** | **PC 2** | **PC3** | **PC 4** |
| --- | --- | --- | --- | --- | --- |
|  | **Eigenvalue** | 35.43 | 17.45 | 12.76 | 3.67 |
|  | **Percent variation** | 43.74 | 21.55 | 15.75 | 4.53 |
|  | **Eigenvectors** |  |  |  |  |
| 1 | Octane | -0.04677 | -0.08064 | -0.24481 | 0.01170 |
| 2 | Acetone | -0.05054 | -0.09173 | 0.18803 | -0.26281 |
| 3 | Isopropyl alcohol | 0.13372 | -0.06828 | 0.13574 | 0.05230 |
| 4 | 2-Butanol | 0.14359 | 0.04420 | -0.00388 | 0.01087 |
| 6 | Ethyl 2-methylbutanoate | 0.15264 | 0.03687 | 0.00988 | 0.01409 |
| 8 | Ethyl isovalerate | 0.14407 | 0.03732 | 0.00409 | 0.02390 |
| 9 | Dimethyl disulfide | -0.07415 | 0.18305 | 0.04460 | 0.21425 |
| 13 | Isoamyl acetate/Pentyl isobutyrate^2^ | 0.14978 | 0.04734 | -0.00317 | -0.00643 |
| 17 | 4-Methyl-2-pentanol | -0.04748 | -0.08220 | -0.24899 | 0.01823 |
| 19 | 2-Heptanone | -0.08528 | -0.00800 | 0.21620 | 0.10227 |
| 20 | Isoamyl propionate | 0.11433 | 0.03727 | -0.00176 | -0.01827 |
| 22 | Isoamyl alcohol/2-methyl-1-butanol^2^ | 0.15619 | 0.06778 | 0.03702 | 0.00787 |
| 24 | 2-Pentylfuran | -0.10923 | 0.16933 | -0.00889 | 0.11753 |
| 25 | Ethyl caproate | -0.02507 | -0.04356 | -0.11589 | -0.01937 |
| 26 | 6-Methyl-2-heptanone | -0.02920 | -0.14768 | 0.18293 | 0.08574 |
| 29 | Isobutenylcarbinol | 0.16201 | -0.01973 | 0.05566 | 0.03904 |
| 30 | 3-Octanone | -0.08221 | 0.19380 | 0.05222 | 0.04102 |
| 31 | Isoamyl butyrate | 0.13019 | 0.04004 | -0.00357 | 0.01083 |
| 32 | Ethenylbenzene | -0.14482 | 0.08010 | 0.07028 | 0.13310 |
| 33 | 2-Propenylbenzene | 0.16343 | 0.05058 | -0.00417 | 0.00743 |
| 37 | 2-Octanone | -0.08003 | 0.18878 | 0.05297 | 0.07715 |
| 39 | Trans-2-(2-Pentenyl)furan | -0.03738 | -0.06639 | -0.19990 | 0.03347 |
| 40 | Acetoin | -0.08372 | 0.08640 | 0.13561 | -0.15032 |
| 42 | 2-Heptanol | -0.06398 | -0.21147 | 0.00517 | 0.09252 |
| 43 | Prenol | 0.16270 | 0.05048 | -0.00406 | 0.00553 |
| 45 | 4-Pentenyl butyrate | 0.11262 | 0.03381 | -0.00362 | 0.02015 |
| 46 | 6-Methyl-5-hepten-2-one | -0.11946 | 0.11231 | 0.10585 | 0.04528 |
| 48 | 1-Hexanol | 0.07408 | 0.13593 | -0.00890 | 0.11516 |
| 49 | 2-Isopropyl pyrazine | -0.02698 | -0.14403 | 0.16870 | 0.04759 |
| 50 | 6-Methyl-2-heptanol | -0.03067 | -0.15982 | 0.18944 | 0.06915 |
| 54 | 3-Octanol | -0.07939 | 0.17173 | 0.05855 | -0.23590 |
| 55 | 2-Nonanone | -0.09076 | 0.01996 | 0.20568 | 0.10052 |
| 57 | Dimethyl trisulfide | -0.08380 | 0.19571 | 0.05431 | 0.01106 |
| 59 | 1-Nitropentane | -0.07540 | 0.18553 | 0.04412 | 0.18214 |
| 62 | Perillene | 0.16160 | 0.04977 | -0.00428 | 0.01050 |
| 64 | Ethyl octanoate | -0.02431 | -0.05734 | -0.10014 | -0.01345 |
| 65 | 2-Methoxy-3-(methylethyl)-pyrazine | -0.03078 | -0.16057 | 0.19369 | 0.07050 |
| 67 | 1-Octen-3-ol | -0.10340 | 0.17462 | -0.01721 | -0.05954 |
| 68 | Linalool oxide | 0.16314 | 0.05055 | -0.00412 | 0.00653 |
| 69 | 1-Heptanol | -0.00325 | 0.15377 | 0.04674 | -0.30279 |
| 70 | 6-Methyl-5-hepten-2-ol | -0.00061 | -0.09691 | 0.16899 | -0.10118 |
| 72 | 2-Ethyl-1-hexanol | -0.08373 | 0.18587 | 0.05949 | -0.15966 |
| 73 | Copaene | 0.16299 | 0.05044 | -0.00418 | 0.00776 |
| 74 | 2,5-Bis(1-methylethyl)pyrazine | -0.03310 | -0.12600 | 0.16738 | -0.05739 |
| 76 | 1-Nitrohexane | -0.07319 | 0.18258 | 0.04210 | 0.23004 |
| 78 | 2-Nonanol | 0.16087 | 0.00988 | 0.04849 | 0.03409 |
| 79 | 3,3-Dimethylcyclohexanol | -0.04755 | -0.08209 | -0.24871 | 0.01647 |
| 80 | 2-Isobutyl-3-methoxypyrazine | -0.03106 | -0.16138 | 0.19518 | 0.07378 |
| 81 | Linalool | 0.16304 | 0.05071 | -0.00399 | 0.00402 |
| 83 | Dihydro-2-methyl-3(2H)-thiophenone | -0.08016 | 0.18278 | 0.05800 | 0.19117 |
| 84 | 1-Octanol | 0.09475 | 0.02253 | 0.15913 | 0.17946 |
| 88 | 2-Undecanone | -0.07467 | 0.16896 | 0.05382 | -0.04634 |
| 91 | Benzonitrile | -0.03038 | -0.15481 | 0.18636 | 0.08233 |
| 92 | Methyl benzoate | 0.16149 | 0.05058 | -0.00375 | -0.00010 |
| 94 | 1-Nonanol | 0.15896 | -0.00954 | 0.07189 | 0.02560 |
| 95 | Isopropyl benzoate | 0.11998 | 0.03713 | -0.00297 | 0.00362 |
| 96 | Acetophenone | -0.13003 | 0.12131 | 0.01490 | -0.15785 |
| 97 | Ethyl benzoate | 0.16375 | 0.04149 | 0.01727 | -0.00506 |
| 99 | Butanoic acid | 0.14249 | 0.04555 | -0.00272 | -0.01216 |
| 102 | 3-(Methylthio)-1-propanol | -0.08292 | 0.19658 | 0.05246 | 0.06703 |
| 103 | Benzyl acetate | 0.15932 | 0.05010 | -0.00357 | -0.00281 |
| 106 | Propyl benzoate | 0.13626 | 0.04221 | -0.00338 | 0.00423 |
| 109 | Methyl salicylate | 0.16426 | 0.04613 | 0.00231 | 0.00505 |
| 110 | 2-Tridecanone | -0.06236 | 0.13365 | 0.05306 | 0.08716 |
| 111 | Phenethyl acetate | 0.14994 | 0.04746 | -0.00313 | -0.00726 |
| 112 | Ethyl salicylate | 0.15832 | 0.00126 | 0.07748 | -0.05722 |
| 113 | 1-Phenyl-2-propanol | -0.04759 | -0.08210 | -0.24891 | 0.01501 |
| 114 | Geranylacetone | -0.08464 | 0.19725 | 0.05651 | 0.02653 |
| 115 | Guaiacol | 0.00137 | -0.11956 | 0.23990 | -0.04408 |
| 116 | Benzyl alcohol | 0.16292 | 0.05430 | -0.00620 | -0.00140 |
| 118 | Phenylethyl alcohol | 0.16378 | 0.04946 | -0.00745 | 0.01317 |
| 119 | Phenethyl butyrate | 0.11258 | 0.03362 | -0.00372 | 0.02221 |
| 120 | Creosol | -0.09441 | -0.12407 | -0.14379 | -0.11643 |
| 123 | Phenol | 0.10081 | -0.10312 | 0.18624 | 0.04634 |
| 124 | 4-Ethylguaiacol | 0.04360 | -0.09373 | 0.18112 | -0.03852 |
| 125 | Benzenepropanol | 0.16293 | 0.05035 | -0.00420 | 0.00832 |
| 126 | 4-Methylphenol | 0.00477 | 0.01108 | 0.11223 | -0.46092 |
| 127 | Eugenol | -0.05468 | -0.16899 | -0.04624 | 0.07510 |
| 128 | 4-Ethylphenol | 0.16207 | 0.05020 | -0.00409 | 0.00650 |
| 129 | 2-Methoxy-4-vinylphenol | -0.01314 | 0.13879 | 0.07158 | -0.33176 |
| 130 | Ethyl palmitate | 0.16109 | 0.05028 | -0.00382 | 0.00155 |

^1^The compounds present only once across all cactus-microorganism combinations were excluded from the PCA. ^2^Compounds co-eluting at the same time on the column.

**Table S10.** Principal component values for volatile compounds for all four host cacti inoculated with the yeast *Pichia cactophila*^1^.

|  |  | **PC 1** | **PC 2** | **PC3** | **PC 4** |
| --- | --- | --- | --- | --- | --- |
|  | **Eigenvalue** | 41.85 | 15.45 | 9.19 | 4.65 |
|  | **Percent variation** | 52.98 | 19.56 | 11.63 | 5.89 |
|  | **Eigenvectors** |  |  |  |  |
| 3 | Isopropyl alcohol | 0.15394 | 0.00860 | 0.02488 | 0.00076 |
| 4 | 2-Butanol | 0.09732 | -0.00024 | 0.00666 | -0.21195 |
| 5 | Ethyl butanoate | 0.12345 | 0.01171 | 0.02847 | 0.21015 |
| 8 | Ethyl isovalerate | 0.13782 | 0.00620 | 0.01698 | -0.15908 |
| 9 | Dimethyl disulfide | -0.05899 | 0.20533 | 0.09325 | -0.03227 |
| 13 | Isoamyl acetate/Pentyl isobutyrate^2^ | -0.03572 | 0.21864 | 0.06257 | 0.04515 |
| 18 | Pentyl acetate | -0.04617 | 0.21880 | 0.05732 | 0.03229 |
| 19 | 2-Heptanone | -0.05833 | -0.16367 | 0.19959 | 0.03601 |
| 22 | Isoamyl alcohol/2-methyl-1-butanol^2^ | 0.14810 | -0.00695 | 0.05251 | -0.08025 |
| 23 | 2-Penten-1-ol, acetate, (Z)- | -0.04603 | 0.21868 | 0.05634 | 0.03246 |
| 24 | 2-Pentylfuran | -0.06322 | 0.12498 | 0.07571 | -0.06769 |
| 25 | Ethyl caproate | 0.15388 | 0.00884 | 0.02534 | 0.01280 |
| 26 | 6-Methyl-2-heptanone | -0.05613 | -0.16711 | 0.13501 | 0.04392 |
| 29 | Isobutenylcarbinol | 0.14903 | -0.01951 | 0.05924 | -0.04605 |
| 30 | 3-Octanone | -0.08510 | 0.09759 | 0.20462 | 0.02686 |
| 31 | Isoamyl butyrate | 0.11044 | 0.01125 | 0.02942 | 0.31393 |
| 32 | Ethenylbenzene | -0.05289 | -0.16438 | 0.19464 | 0.03641 |
| 33 | 2-Propenylbenzene | 0.15315 | 0.00799 | 0.02397 | -0.01607 |
| 34 | Hexyl acetate | -0.04665 | 0.21919 | 0.05635 | 0.03203 |
| 37 | 2-Octanone | -0.08624 | -0.04419 | 0.23421 | -0.01626 |
| 39 | Trans-2-(2-Pentenyl)furan | -0.03592 | -0.04523 | -0.28739 | 0.03891 |
| 40 | Acetoin | -0.05352 | -0.13306 | 0.19704 | 0.00825 |
| 43 | Prenol | 0.15370 | 0.00815 | 0.02376 | -0.02940 |
| 45 | 4-Pentenyl butyrate | 0.10395 | 0.01111 | 0.02901 | 0.33228 |
| 46 | 6-Methyl-5-hepten-2-one | -0.09622 | -0.16486 | -0.09858 | 0.06721 |
| 48 | 1-Hexanol | 0.14797 | 0.02343 | 0.03958 | -0.08206 |
| 51 | Heptyl acetate | -0.04610 | 0.21780 | 0.05827 | 0.03190 |
| 55 | 2-Nonanone | -0.08587 | -0.11342 | 0.22000 | 0.00193 |
| 57 | Dimethyl trisulfide | -0.04989 | 0.13093 | 0.09181 | -0.06903 |
| 60 | 2-Hexen-1-ol | 0.15144 | 0.00888 | 0.02420 | -0.01751 |
| 62 | Perillene | 0.15396 | 0.00865 | 0.02481 | -0.00299 |
| 64 | Ethyl octanoate | 0.14918 | 0.00874 | 0.02342 | -0.03206 |
| 65 | 2-Methoxy-3-(methylethyl)-pyrazine | -0.05390 | -0.16866 | 0.19031 | 0.03897 |
| 68 | Linalool oxide | 0.15240 | 0.00750 | 0.02217 | -0.06709 |
| 69 | 1-Heptanol | 0.15072 | 0.00717 | 0.02107 | -0.09218 |
| 70 | 6-Methyl-5-hepten-2-ol | 0.11790 | 0.12659 | 0.12898 | -0.03279 |
| 71 | Octyl acetate | -0.04735 | 0.22095 | 0.05935 | 0.02956 |
| 72 | 2-Ethyl-1-hexanol | -0.05407 | -0.16911 | 0.18934 | 0.03931 |
| 73 | Copaene | 0.15221 | -0.00893 | 0.03039 | -0.06189 |
| 76 | 1-Nitrohexane | -0.05769 | 0.18739 | 0.09499 | -0.04477 |
| 78 | 2-Nonanol | 0.15355 | 0.00912 | 0.02554 | 0.01582 |
| 80 | 2-Isobutyl-3-methoxypyrazine | -0.05412 | -0.16909 | 0.19230 | 0.03880 |
| 81 | Linalool | 0.15093 | 0.00687 | 0.02099 | -0.09020 |
| 82 | Benzaldehyde | -0.06083 | -0.10445 | 0.20291 | -0.02799 |
| 83 | Dihydro-2-methyl-3(2H)-thiophenone | -0.06418 | 0.22865 | 0.04476 | 0.00265 |
| 84 | 1-Octanol | 0.13672 | 0.07721 | 0.06266 | -0.09815 |
| 85 | Nonyl acetate | -0.05272 | 0.22759 | 0.07101 | 0.01466 |
| 88 | 2-Undecanone | -0.09037 | 0.00996 | 0.23890 | -0.02325 |
| 89 | 6-Methyl-3,5-heptadien-2-one | -0.03931 | -0.04957 | -0.30795 | 0.03961 |
| 91 | Benzonitrile | -0.06511 | -0.04853 | 0.19741 | -0.04533 |
| 92 | Methyl benzoate | 0.15282 | 0.00783 | 0.02274 | -0.05528 |
| 93 | 3-(Methylthio)propyl acetate | -0.04666 | 0.21979 | 0.05789 | 0.03116 |
| 94 | 1-Nonanol | 0.15089 | 0.00704 | 0.02101 | -0.09251 |
| 95 | Isopropyl benzoate | 0.15372 | 0.00826 | 0.02433 | -0.01133 |
| 96 | Acetophenone | -0.13523 | 0.04645 | 0.11258 | 0.02496 |
| 97 | Ethyl benzoate | 0.15263 | 0.00943 | 0.02673 | 0.05654 |
| 99 | Butanoic acid | 0.14499 | 0.01045 | 0.02686 | 0.07685 |
| 100 | Ionene | -0.03689 | -0.04653 | -0.29418 | 0.03924 |
| 102 | 3-(Methylthio)-1-propanol | -0.05738 | 0.18314 | 0.09761 | -0.04685 |
| 103 | Benzyl acetate | 0.08718 | 0.18354 | 0.06588 | 0.05833 |
| 105 | Isopropyl phenylacetate | 0.12035 | 0.01005 | 0.02848 | 0.25762 |
| 107 | Methyl phenylacetate | 0.13275 | 0.01018 | 0.02885 | 0.21043 |
| 108 | Ethyl phenylacetate | 0.11103 | 0.01073 | 0.02900 | 0.30631 |
| 109 | Methyl salicylate | 0.15161 | 0.00434 | 0.02622 | -0.05156 |
| 111 | Phenethyl acetate | -0.04796 | 0.21558 | 0.05549 | 0.03517 |
| 112 | Ethyl salicylate | 0.10805 | 0.00475 | 0.01097 | -0.20606 |
| 114 | Geranylacetone | -0.08322 | 0.14080 | -0.15215 | -0.00155 |
| 115 | Guaiacol | 0.15003 | 0.00664 | 0.02117 | -0.07518 |
| 116 | Benzyl alcohol | 0.15285 | 0.00766 | 0.02320 | -0.03595 |
| 118 | Phenylethyl alcohol | 0.04211 | -0.17782 | 0.18231 | -0.04132 |
| 119 | Phenethyl butyrate | 0.10103 | 0.01116 | 0.02888 | 0.34030 |
| 122 | Isoamyl phenylacetate | 0.09687 | 0.01092 | 0.02847 | 0.34975 |
| 123 | Phenol | 0.15115 | 0.00656 | 0.03156 | -0.07536 |
| 124 | 4-Ethylguaiacol | 0.13866 | 0.00456 | 0.01725 | -0.12103 |
| 125 | Benzenepropanol | 0.13929 | 0.00429 | 0.01572 | -0.17340 |
| 126 | 4-Methylphenol | 0.14910 | 0.00765 | 0.02401 | 0.01070 |
| 128 | 4-Ethylphenol | 0.15350 | 0.00809 | 0.02405 | -0.01697 |
| 129 | 2-Methoxy-4-vinylphenol | 0.13907 | 0.01614 | 0.02733 | 0.04905 |
| 130 | Ethyl palmitate | 0.15297 | 0.00949 | 0.02634 | 0.03979 |

^1^The compounds present only once across all cactus-microorganism combinations were excluded from the PCA. ^2^Compounds co-eluting at the same time on the column.

**Table S11.** Principal component values for volatile compounds for all four host cacti inoculated with the yeast *Pichia mexicana*^1^.

|  |  | **PC 1** | **PC 2** | **PC3** | **PC 4** |
| --- | --- | --- | --- | --- | --- |
|  | **Eigenvalue** | 41.53 | 17.90 | 17.01 | 6.48 |
|  | **Percent variation** | 45.64 | 19.67 | 18.69 | 7.12 |
|  | **Eigenvectors** |  |  |  |  |
| 3 | Isopropyl alcohol | 0.14247 | 0.00327 | 0.02234 | -0.12155 |
| 4 | 2-Butanol | 0.13700 | 0.00568 | 0.01939 | -0.12123 |
| 5 | Ethyl butanoate | 0.14194 | 0.00525 | 0.02048 | -0.12011 |
| 8 | Ethyl isovalerate | 0.10807 | 0.00849 | 0.01718 | -0.27577 |
| 9 | Dimethyl disulfide | -0.06120 | -0.12347 | 0.16975 | 0.00147 |
| 13 | Isoamyl acetate/Pentyl isobutyrate^2^ | 0.15276 | -0.00303 | 0.02542 | 0.05159 |
| 14 | 2-Pentanol | -0.06933 | 0.13888 | -0.13083 | 0.01798 |
| 15 | Butanoic acid, 1-methylpropyl ester | 0.11092 | 0.00843 | 0.01510 | -0.19029 |
| 16 | Isobutyl butanoate | 0.15217 | 0.00215 | 0.02300 | -0.06175 |
| 17 | 4-Methyl-2-pentanol | -0.03869 | -0.09585 | -0.21045 | 0.00215 |
| 19 | 2-Heptanone | -0.05856 | 0.21593 | 0.02535 | 0.03070 |
| 21 | 3-Hexanol | -0.03880 | -0.09621 | -0.21125 | 0.00181 |
| 22 | Isoamyl alcohol/2-methyl-1-butanol^2^ | 0.13999 | 0.04728 | -0.00898 | -0.06657 |
| 24 | 2-Pentylfuran | -0.10818 | -0.13337 | 0.10205 | 0.01208 |
| 25 | Ethyl caproate | 0.14763 | -0.00325 | 0.02175 | 0.11482 |
| 26 | 6-Methyl-2-heptanone | -0.05111 | 0.21894 | 0.00804 | 0.02590 |
| 27 | Prenyl acetate | 0.11521 | -0.00456 | 0.01936 | 0.06881 |
| 29 | Isobutenylcarbinol | 0.04176 | 0.18577 | -0.00905 | -0.10933 |
| 30 | 3-Octanone | -0.06100 | -0.12449 | 0.17783 | -0.00185 |
| 31 | Isoamyl butyrate | 0.12394 | 0.00538 | 0.02006 | -0.20104 |
| 32 | Ethenylbenzene | -0.05827 | 0.20402 | -0.00890 | 0.03614 |
| 33 | 2-Propenylbenzene | 0.13530 | -0.00088 | 0.01831 | 0.09816 |
| 36 | 5-Methyl-2-hexanol | -0.03805 | -0.09389 | -0.20632 | 0.00291 |
| 37 | 2-Octanone | -0.09469 | 0.10145 | 0.14892 | 0.03026 |
| 39 | Trans-2-(2-Pentenyl)furan | -0.03872 | -0.09566 | -0.21056 | 0.00195 |
| 40 | Acetoin | -0.03842 | 0.15236 | 0.00603 | 0.03550 |
| 41 | Hexanenitrile | -0.06107 | -0.12299 | 0.17473 | 0.00161 |
| 42 | 2-Heptanol | -0.03395 | -0.04621 | -0.22899 | 0.01243 |
| 43 | Prenol | 0.15232 | 0.00176 | 0.02311 | -0.05346 |
| 45 | 4-Pentenyl butyrate | 0.08667 | 0.00928 | 0.01441 | -0.31456 |
| 46 | 6-Methyl-5-hepten-2-one | -0.09831 | 0.17756 | 0.01925 | 0.03543 |
| 48 | 1-Hexanol | -0.00271 | -0.08496 | 0.21578 | -0.01992 |
| 50 | 6-Methyl-2-heptanol | -0.06278 | 0.14282 | -0.10983 | 0.01485 |
| 54 | 3-Octanol | -0.07951 | -0.17438 | 0.03243 | -0.00326 |
| 55 | 2-Nonanone | -0.07382 | 0.19542 | 0.06476 | 0.03489 |
| 57 | Dimethyl trisulfide | -0.04629 | -0.09054 | 0.12298 | 0.00704 |
| 59 | 1-Nitropentane | -0.09330 | 0.03393 | 0.17833 | 0.02374 |
| 62 | Perillene | 0.14435 | 0.00486 | 0.02173 | -0.13827 |
| 63 | Benzene,m-di-tert-butyl | -0.05988 | -0.12161 | 0.16708 | -0.00028 |
| 64 | Ethyl octanoate | 0.15274 | -0.00134 | 0.02294 | 0.04494 |
| 65 | 2-Methoxy-3-(methylethyl)-pyrazine | -0.05292 | 0.21775 | 0.01368 | 0.03353 |
| 67 | 1-Octen-3-ol | -0.09040 | 0.13706 | 0.12692 | 0.02962 |
| 68 | Linalool oxide | 0.15328 | 0.00175 | 0.02302 | -0.04517 |
| 69 | 1-Heptanol | -0.06067 | -0.12365 | 0.17789 | -0.00156 |
| 70 | 6-Methyl-5-hepten-2-ol | -0.05490 | -0.02611 | 0.12983 | -0.08901 |
| 72 | 2-Ethyl-1-hexanol | -0.04429 | 0.04944 | 0.21598 | -0.02517 |
| 73 | Copaene | 0.14436 | 0.00448 | 0.02201 | -0.13668 |
| 75 | Methyl 2-hydroxy-3-methyl pentanoate | -0.05184 | 0.22018 | 0.00962 | 0.02740 |
| 76 | 1-Nitrohexane | -0.05736 | -0.11742 | 0.17185 | -0.00267 |
| 78 | 2-Nonanol | 0.15417 | 0.00043 | 0.02313 | -0.00611 |
| 79 | 3,3-Dimethylcyclohexanol | -0.03852 | -0.09595 | -0.21043 | 0.00097 |
| 80 | 2-Isobutyl-3-methoxypyrazine | -0.05262 | 0.22094 | 0.01101 | 0.02995 |
| 81 | Linalool | 0.15406 | 0.00678 | 0.02344 | -0.02241 |
| 82 | Benzaldehyde | -0.08375 | 0.02472 | 0.16008 | 0.03213 |
| 83 | Dihydro-2-methyl-3(2H)-thiophenone | -0.06355 | -0.13014 | 0.17274 | -0.00148 |
| 84 | 1-Octanol | 0.06810 | 0.13676 | 0.12605 | -0.05289 |
| 86 | Acetic acid | 0.10880 | -0.00857 | 0.01710 | 0.22914 |
| 88 | 2-Undecanone | -0.05148 | 0.21147 | 0.01071 | 0.03550 |
| 90 | 4-Terpineol | -0.04725 | 0.20794 | 0.00519 | 0.01881 |
| 91 | Benzonitrile | -0.07950 | 0.17735 | 0.08416 | 0.03754 |
| 92 | Methyl benzoate | 0.14308 | -0.00362 | 0.02054 | 0.14287 |
| 94 | 1-Nonanol | 0.12763 | 0.09620 | 0.03106 | -0.09977 |
| 95 | Isopropyl benzoate | 0.12482 | -0.00461 | 0.01694 | 0.20004 |
| 96 | Acetophenone | -0.13422 | -0.05780 | 0.09893 | 0.02323 |
| 97 | Ethyl benzoate | 0.13076 | -0.00519 | 0.01835 | 0.19975 |
| 99 | Butanoic acid | 0.13915 | -0.00520 | 0.02068 | 0.16451 |
| 102 | 3-(Methylthio)-1-propanol | -0.06188 | -0.12590 | 0.17754 | -0.00098 |
| 103 | Benzyl acetate | 0.15113 | -0.00218 | 0.02239 | 0.07963 |
| 104 | sec-Butyl benzoate | 0.12668 | -0.00569 | 0.01769 | 0.21571 |
| 106 | Propyl benzoate | 0.12736 | -0.00590 | 0.01794 | 0.21699 |
| 109 | Methyl salicylate | 0.15372 | 0.00861 | 0.02348 | -0.03304 |
| 110 | 2-Tridecanone | -0.04536 | 0.19472 | 0.00170 | 0.02756 |
| 111 | Phenethyl acetate | 0.14555 | -0.00382 | 0.02149 | 0.12975 |
| 112 | Ethyl salicylate | 0.15310 | 0.00167 | 0.02308 | -0.04576 |
| 113 | 1-Phenyl-2-propanol | -0.03866 | -0.09623 | -0.21097 | 0.00131 |
| 114 | Geranylacetone | -0.06134 | -0.12352 | 0.17440 | 0.00168 |
| 115 | Guaiacol | 0.04631 | 0.12802 | 0.00007 | -0.06560 |
| 116 | Benzyl alcohol | 0.15203 | 0.00609 | 0.03639 | -0.04819 |
| 117 | Pentyl benzoate | 0.12669 | -0.00570 | 0.01770 | 0.21592 |
| 118 | Phenylethyl alcohol | 0.12251 | 0.13543 | 0.01096 | -0.04908 |
| 119 | Phenethyl butyrate | 0.07512 | 0.00993 | 0.01269 | -0.33446 |
| 120 | Creosol | -0.04360 | -0.07454 | -0.21521 | 0.00064 |
| 121 | 3-Methyl-3-butenyl benzoate | 0.12678 | -0.00601 | 0.01787 | 0.21971 |
| 123 | Phenol | 0.11203 | 0.08903 | -0.00153 | -0.06868 |
| 124 | 4-Ethylguaiacol | 0.15386 | -0.00063 | 0.02284 | 0.03327 |
| 125 | Benzenepropanol | 0.15413 | 0.00125 | 0.02287 | -0.02127 |
| 126 | 4-Methylphenol | 0.09562 | -0.00868 | 0.01587 | 0.20681 |
| 127 | Eugenol | -0.03879 | -0.09591 | -0.21112 | 0.00167 |
| 128 | 4-Ethylphenol | 0.15204 | -0.00167 | 0.02288 | 0.05300 |
| 129 | 2-Methoxy-4-vinylphenol | 0.10825 | -0.10126 | 0.03778 | 0.02527 |
| 130 | Ethyl palmitate | 0.15399 | -0.00037 | 0.02275 | 0.02914 |

^1^The compounds present only once across all cactus-microorganism combinations were excluded from the PCA. ^2^Compounds co-eluting at the same time on the column.

**Table S12.** Analysis of variance for each measurement. (a-e) Whole model and population specific ANOVAs for total viability, pupal viability, developmental time, thorax length and body weight, respectively.

| **(a) Total Viability** | | | | | |
| --- | --- | --- | --- | --- | --- |
| **Whole model ANOVA** | | | | | |
| **Source of variation** | **d.f.** | **SS** | **F** | ***P*** |  |
| P | 3 | 1020.49 | 3.63 | 0.0232 |  |
| C | 3 | 18508.11 | 65.86 | < 0.0001 |  |
| P X C | 9 | 4360.13 | 5.17 | 0.0002 |  |
| **Population specific ANOVAs** | | | | | |
| **Source of variation** | **d.f.** | **SS** | **MS** | **F** | ***P*** |
| **Mojave** | | | | | |
| Model | 3 | 12483.67 | 4161.22 | 33.65 | < 0.0001 |
| Error | 8 | 989.33 | 123.67 |  |  |
| C. Total | 11 | 13473.00 |  |  |  |
| **S. Catalina** | | | | | |
| Model | 3 | 2741.30 | 913.77 | 12.23 | 0.0023 |
| Error | 8 | 597.86 | 74.73 |  |  |
| C. Total | 11 | 3339.16 |  |  |  |
| **mainland Sonoran** | | | | | |
| Model | 3 | 5267.20 | 1755.73 | 25.78 | 0.0002 |
| Error | 8 | 544.81 | 68.10 |  |  |
| C. Total | 11 | 5812.01 |  |  |  |
| **Baja** | | | | | |
| Model | 3 | 2376.07 | 792.02 | 7.32 | 0.0111 |
| Error | 8 | 865.74 | 108.22 |  |  |
| C. Total | 11 | 3241.81 |  |  |  |
| **(b) Pupal Viability** | | | | | |
| **Whole model ANOVA** | | | | | |
| **Source of variation** | **d.f.** | **SS** | **F** | ***P*** |  |
| P | 3 | 3350.92 | 23.85 | < 0.0001 |  |
| C | 3 | 21426.92 | 152.50 | < 0.0001 |  |
| P X C | 9 | 3408.75 | 8.09 | < 0.0001 |  |
| **Population specific ANOVAs** | | | | | |
| **Source of variation** | **d.f.** | **SS** | **MS** | **F** | ***P*** |
| **Mojave** |  |  |  |  |  |
| Model | 3 | 13886.33 | 4628.78 | 204.21 | < 0.0001 |
| Error | 8 | 181.33 | 2.67 |  |  |
| C. Total | 11 | 14067.67 |  |  |  |
| **S. Catalina** |  |  |  |  |  |
| Model | 3 | 3090.67 | 1030.22 | 16.98 | 0.0008 |
| Error | 8 | 485.33 | 60.67 |  |  |
| C. Total | 11 | 3576.00 |  |  |  |
| **mainland Sonoran** |  |  |  |  |  |
| Model | 3 | 4486.67 | 1495.56 | 35.0521 | < 0.0001 |
| Error | 8 | 341.33 | 42.67 |  |  |
| C. Total | 11 | 4828.00 |  |  |  |
| **Baja** |  |  |  |  |  |
| Model | 3 | 3372.00 | 1124.00 | 18.33 | 0.0006 |
| Error | 8 | 490.67 | 61.33 |  |  |
| C. Total | 11 | 3862.67 |  |  |  |
| **(c) Developmental time** | | | | | |
| **Whole model ANOVA** | | | | | |
| **Source of variation** | **d.f.** | **SS** | **F** | ***P*** |  |
| P | 3 | 1209250.00 | 80.79 | < 0.0001 |  |
| C | 3 | 18542589.00 | 1238.76 | < 0.0001 |  |
| S | 1 | 4102.00 | 0.82 | 0.3647 |  |
| P X C | 9 | 1151857.00 | 25.65 | < 0.0001 |  |
| P X S | 3 | 22106.00 | 1.48 | 0.2190 |  |
| C X S | 3 | 4597.00 | 0.31 | 0.8202 |  |
| P X C X S | 9 | 52711.00 | 1.17 | 0.3076 |  |
| **Population specific ANOVAs** | | | | | |
| **Source of variation** | **d.f.** | **SS** | **F** | ***P*** |  |
| **Mojave** | | | | | |
| C | 3 | 6956674.40 | 426.97 | < 0.0001 |  |
| S | 1 | 887.10 | 0.16 | 0.6864 |  |
| C X S | 3 | 10730.30 | 0.66 | 0.5781 |  |
| **S. Catalina** | | | | | |
| C | 3 | 3351199.60 | 581.07 | < 0.0001 |  |
| S | 1 | 4667.30 | 2.43 | 0.1200 |  |
| C X S | 3 | 10526.3 | 1.83 | 0.1419 |  |
| **mainland Sonoran** | | | | | |
| C | 3 | 5021368.10 | 348.07 | < 0.0001 |  |
| S | 1 | 18842.50 | 3.92 | 0.0484 |  |
| C X S | 3 | 23544.20 | 1.63 | 0.1814 |  |
| **Baja** | | | | | |
| C | 3 | 5376184.80 | 225.71 | < 0.0001 |  |
| S | 1 | 2648.10 | 0.33 | 0.5639 |  |
| C X S | 3 | 17665.30 | 0.74 | 0.5277 |  |
| **Sex specific ANOVAs** | | | | | |
| **Source of variation** | **d.f.** | **SS** | **MS** | **F** | ***P*** |
| **mainland Sonoran Male** | | | | | |
| Model | 3 | 2293161.30 | 764387 | 154.64 | < 0.0001 |
| Error | 203 | 1003411.90 | 4943 |  |  |
| C. Total | 206 | 3296573.20 |  |  |  |
| **mainland Sonoran Female** | | | | | |
| Model | 3 | 2831443.50 | 943815 | 201.93 | < 0.0001 |
| Error | 202 | 944144.20 | 4674 |  |  |
| C. Total | 205 | 3775587.70 |  |  |  |
| **(d) Thorax length** | | | | | |
| **Whole model ANOVA** | | | | | |
| **Source of variation** | **d.f.** | **SS** | **F** | ***P*** |  |
| P | 3 | 0.85 | 155.83 | < 0.0001 |  |
| C | 3 | 2.28 | 419.18 | < 0.0001 |  |
| S | 1 | 0.36 | 199.38 | < 0.0001 |  |
| P X C | 9 | 0.08 | 4.71 | < 0.0001 |  |
| P X S | 3 | 0.02 | 3.54 | 0.0145 |  |
| C X S | 3 | 0.02 | 3.39 | 0.0180 |  |
| P X C X S | 9 | 0.02 | 1.16 | 0.3196 |  |
| **Population specific ANOVAs** | | | | | |
| **Source of variation** | **d.f.** | **SS** | **F** | ***P*** |  |
| **Mojave** | | | | | |
| C | 3 | 0.54 | 88.01 | < 0.0001 |  |
| S | 1 | 0.04 | 21.75 | < 0.0001 |  |
| C X S | 3 | 0.02 | 2.95 | 0.0351 |  |
| **S. Catalina** | | | | | |
| C | 3 | 0.65 | 174.71 | < 0.0001 |  |
| S | 1 | 0.07 | 52.88 | < 0.0001 |  |
| C X S | 3 | 0.01 | 290 | 0.0370 |  |
| **mainland Sonoran** | | | | | |
| C | 3 | 0.48 | 102.68 | < 0.0001 |  |
| S | 1 | 0.22 | 140.16 | < 0.0001 |  |
| C X S | 3 | 0.00 | 1.00 | 0.3927 |  |
| **Baja** | | | | | |
| C | 3 | 0.70 | 95.05 | < 0.0001 |  |
| S | 1 | 0.18 | 74.74 | < 0.0001 |  |
| C X S | 3 | 0.01 | 0.81 | 0.4890 |  |
| **Sex specific ANOVAs** | | | | | |
| **Source of variation** | **d.f.** | **SS** | **MS** | **F** | ***P*** |
| **S. Catalina Male** | | | | | |
| Model | 3 | 0.27 | 0.09 | 65.38 | < 0.0001 |
| Error | 68 | 0.09 | 0.00 |  |  |
| C. Total | 71 | 0.36 |  |  |  |
| **S. Catalina Female** | | | | | |
| Model | 3 | 0.40 | 0.13 | 118.44 | < 0.0001 |
| Error | 76 | 0.08 | 0.00 |  |  |
| C. Total | 79 | 0.48 |  |  |  |
| **(e) Body Weight** | | | | | |
| **Whole model ANOVA** | | | | | |
| **Source of variation** | **d.f.** | **SS** | **F** | ***P*** |  |
| P | 3 | 1.87 | 444.50 | < 0.0001 |  |
| C | 3 | 4.97 | 1181.65 | < 0.0001 |  |
| S | 1 | 0.24 | 170.46 | < 0.0001 |  |
| P X C | 9 | 0.30 | 23.54 | < 0.0001 |  |
| P X S | 3 | 0.07 | 15.70 | < 0.0001 |  |
| C X S | 3 | 0.08 | 18.53 | < 0.0001 |  |
| P X C X S | 9 | 0.04 | 2.93 | 0.0020 |  |
| **Population specific ANOVAs** | | | | | |
| **Source of variation** | **d.f.** | **SS** | **F** | **P** |  |
| **Mojave** | | | | | |
| C | 2 | 1.78 | 684.28 | < 0.0001 |  |
| S | 1 | 0.14 | 109.00 | < 0.0001 |  |
| C X S | 2 | 0.06 | 21.25 | < 0.0001 |  |
| **S. Catalina** | | | | | |
| C | 3 | 0.54 | 344.21 | < 0.0001 |  |
| S | 1 | 0.02 | 44.64 | < 0.0001 |  |
| C X S | 3 | 0.01 | 9.11 | < 0.0001 |  |
| **Mainland Sonoran** | | | | | |
| C | 3 | 1.58 | 312.30 | < 0.0001 |  |
| S | 1 | 0.18 | 106.17 | < 0.0001 |  |
| C X S | 3 | 0.04 | 7.58 | < 0.0001 |  |
| **Baja** | | | | | |
| C | 3 | 1.52 | 244.83 | < 0.0001 |  |
| S | 1 | 0.05 | 25.12 | < 0.0001 |  |
| C X S | 3 | 0.01 | 1.40 | 0.25 |  |
| **Sex specific ANOVAs** | | | | | |
| **Source of variation** | **d.f.** | **SS** | **MS** | **F** | ***P*** |
| **Mojave Male** | | | | | |
| Model | 2 | 0.67 | 0.34 | 547.18 | < 0.0001 |
| Error | 80 | 0.05 | 0.00 |  |  |
| C. Total | 82 | 0.72 |  |  |  |
| **Mojave Female** | | | | | |
| Model | 2 | 1.78 | 0.59 | 298.44 | < 0.0001 |
| Error | 81 | 0.16 | 0.00 |  |  |
| C. Total | 83 | 1.34 |  |  |  |
| **S. Catalina Male** | | | | | |
| Model | 3 | 0.21 | 0.07 | 190.37 | < 0.0001 |
| Error | 111 | 0.04 | 0.00 |  |  |
| C. Total | 114 | 0.26 |  |  |  |
| **S. Catalina Female** | | | | | |
| Model | 3 | 0.33 | 0.11 | 160.38 | < 0.0001 |
| Error | 100 | 0.07 | 0.00 |  |  |
| C. Total | 103 | 0.40 |  |  |  |
| **mainland Sonoran Male** | | | | | |
| Model | 3 | 0.55 | 0.18 | 140.81 | < 0.0001 |
| Error | 111 | 0.15 | 0.00 |  |  |
| C. Total | 114 | 0.70 |  |  |  |
| **mainland Sonoran Female** | | | | | |
| Model | 3 | 1.09 | 0.36 | 172.08 | < 0.0001 |
| Error | 101 | 0.21 | 0.00 |  |  |
| C. Total | 104 | 1.30 |  |  |  |

**Table S13.** Relative performance indices for each *D. mojavensis* population on all four host cacti.

|  | **Mojave** | **S. Catalina** | **Sonoran** | **Baja** |
| --- | --- | --- | --- | --- |
| Barrel | 0.1361 | 0.0806 | 0.1126 | 0.0994 |
| Prickly Pear | 0.0631 | 0.0325 | 0.0524 | 0.0515 |
| Agria | 0.0187 | 0.0159 | 0.0332 | 0.0238 |
| Organ Pipe | 0.0033 | 0.0169 | 0.0148 | 0.0155 |
